# New subfamilies of major intrinsic proteins in fungi suggest novel transport properties in fungal channels: implications for the host-fungal interactions

**DOI:** 10.1186/s12862-014-0173-4

**Published:** 2014-08-12

**Authors:** Ravi Kumar Verma, Neel Duti Prabh, Ramasubbu Sankararamakrishnan

**Affiliations:** 1 Department of Biological Sciences and Bioengineering, Indian Institute of Technology Kanpur, Kanpur 208016, India; 2 Center of Excellence for Chemical Biology, Indian Institute of Technology Kanpur, Kanpur 208016, India

**Keywords:** Aquaglyceroporin, Aquaporin, Aromatic/arginine selectivity filter, Host-pathogen interactions, Intra-helical salt-bridge, Membrane transport, Plant-fungi symbiosis, Selective transport

## Abstract

**Background:**

Aquaporins (AQPs) and aquaglyceroporins (AQGPs) belong to the superfamily of Major Intrinsic Proteins (MIPs) and are involved in the transport of water and neutral solutes across the membranes. MIP channels play significant role in plant-fungi symbiotic relationship and are believed to be important in host-pathogen interactions in human fungal diseases. In plants, at least five major MIP subfamilies have been identified. Fungal MIP subfamilies include orthodox aquaporins and five subgroups within aquaglyceroporins. XIP subfamily is common to both plants and fungi. In this study, we have investigated the extent of diversity in fungal MIPs and explored further evolutionary relationships with the plant MIP counterparts.

**Results:**

We have extensively analyzed the available fungal genomes and examined nearly 400 fungal MIPs. Phylogenetic analysis and homology modeling exhibit the existence of a new MIP cluster distinct from any of the known fungal MIP subfamilies. All members of this cluster are found in microsporidia which are unicellular fungal parasites. Members of this family are small in size, charged and have hydrophobic residues in the aromatic/arginine selectivity filter and these features are shared by small and basic intrinsic proteins (SIPs), one of the plant MIP subfamilies. We have also found two new subfamilies (δ and γ2) within the AQGP group. Fungal AQGPs are the most diverse and possess the largest number of subgroups. We have also identified distinguishing features in loops E and D in the newly identified subfamilies indicating their possible role in channel transport and gating.

**Conclusions:**

Fungal SIP-like MIP family is distinct from any of the known fungal MIP families including orthodox aquaporins and aquaglyceroporins. After XIPs, this is the second MIP subfamily from fungi that may have possible evolutionary link with a plant MIP subfamily. AQGPs in fungi are more diverse and possess the largest number of subgroups. The aromatic/arginine selectivity filter of SIP-like fungal MIPs and the δ AQGPs are unique, hydrophobic in nature and are likely to transport novel hydrophobic solutes. They can be attractive targets for developing anti-fungal drugs. The evolutionary pattern shared with their plant counterparts indicates possible involvement of new fungal MIPs in plant-fungi symbiosis and host-pathogen interactions.

## Background

The superfamily of Major Intrinsic Proteins (MIPs) contains channel proteins that transport water and other neutral solutes [1]–[4]. These integral membrane proteins are found from bacteria to humans and are abundantly present in plants [5],[6]. Aquaporin (AQP) and aquaglyceroporin (AQGP) are the prototype members of the MIP superfamily [7],[8]. While in mammals three major subfamilies are detected [9], plant MIPs are found to have at least five subfamilies [10]–[12]. In humans, MIP members play significant role in kidney nephron, epithelial fluid secretion, maintaining brain water balance, cell migration, skin hydration, adipocyte metabolism and neuroexcitation [9],[13]–[15]. They are implicated in various human diseases such as glaucoma, epilepsy, cancer, obesity, nephrogenic diabetes insipidus and neuromyelitis optica [15]–[22]. In plants, multiple roles have been recognized in terms of plant development, growth and physiology [2],[23],[24]. They have been shown to be important in stress tolerance in plants and they play vital role in their interactions with soil microorganisms, fungi and pathogens [25]–[29]. In addition to water and glycerol, expression studies in *Xenopus* oocytes showed that MIPs transport diverse neutral solutes and gases such as ammonia and CO_2_[1],[3],[25]. MIP members also serve as an important component in host-parasite interactions and several protozoan parasite aquaporins have been identified including those from *Plasmodium*, *Trypanosoma* and *Leishmania* species [30]–[34]. Functional studies suggest that protozoan aquaporins are likely to transport both water and glycerol efficiently [31]. These parasite channel proteins could either serve as potential drug targets or vehicles for transporting cytotoxic compounds [30],[31],[35]. We have recently identified more than 1000 MIP genes from more than 340 organisms and details about the genes, protein products and structural models are available in MIPModDB database [6].

Symbiotic relationship between plants and fungi occurs through mycorrhiza and these interactions help in translocating soil nutrients to the host plants [36],[37]. This mutualistic association also aids in the transfer of organic carbon from plants to fungal partners [38]. In the mycorrhized plants, movement of water and other nutrients are greatly influenced by the expression of both plant and fungal aquaporins and this plays a major role in the drought resistance of plants [39],[40]. The expression of plant aquaporins in mycorrhized and non-mycorrhized forms have been investigated in several plants under different stress conditions [27],[28],[41]–[43]. On the contrary, very few studies have been carried out on the role of fungal aquaporins in mycorrhizal symbiosis [44],[45]. In addition to their role in root water transport in plants through symplastic pathway, fungal aquaporins present in pathogenic fungal species may act as attractive targets for antifungal drugs [16],[31],[46]. With increasing drug resistance reported in human fungal pathogens [47],[48], it has become even more important to identify new drug targets in fungal organisms.

Fungal aquaporins have been identified in individual species such as *Laccaria bicolor* (an ectomycorrhizal fungus) [44]*Glomus intraradices* (an arbuscular mycorrhizal fungus) [39] and *Encephalitozoon cuniculi* (a microsporidia pathogenic to humans) [49]. Their transport properties, gene expression profiles and role in mycorrhizia or pathogenesis have been investigated. Available literature shows that only a few fungal MIPs have been studied in detail. Fungal species are diverse and form a large group of eukaryotic organisms and hence it is anticipated that the MIP channels present in fungi also could be diverse with different transport specificities and unique regulatory mechanisms and/or physiological functions. In an earlier study, Pettersson et al. [50] analyzed genomes of yeast and filamentous fungi. Their phylogenetic analysis with a small number of 55 MIPs revealed three different subgroups within fungal AQGPs in addition to members belonging to orthodox AQP family. A new subfamily of fungal aquaporins, called X-intrinsic proteins (XIPs), has been discovered in our laboratory which is found to be common between fungi and plants [12]. More recently, Dietz et al. [44] and Zwiazek and coworkers [51] have identified even a larger number of 135 and 229 fungal MIPs respectively from database searches. Both their phylogenetic studies indicated at least four clusters including the orthodox AQPs, XIPs and two to four distinct subgroups of AQGPs. With the improved technologies and falling costs of sequencing, fungal genomes are rapidly sequenced and they are available in public databases. In this study, we have systematically searched completely and partially sequenced fungal genomes and identified a large number of fungal MIPs. With a comprehensive analysis of these MIP sequences using different bioinformatics tools, we have identified a new group of fungal MIPs which forms a distinct cluster separately from the orthodox AQPs and AQGPs. Members of this new family share several features with plant SIP (small and basic intrinsic proteins) subfamily. Our analysis also revealed two new subfamilies of aquaglyceroporins which are separate from the other subgroups of AQGPs. Distinct features of these three new families of fungal MIPs, their possible evolutionary relationship with plants and lower organisms and the nature of solutes that are likely to be transported across these channels are presented in detail.

## Results

### Identification of fungal MIPs and their taxonomic distributions

**Figure 1:**
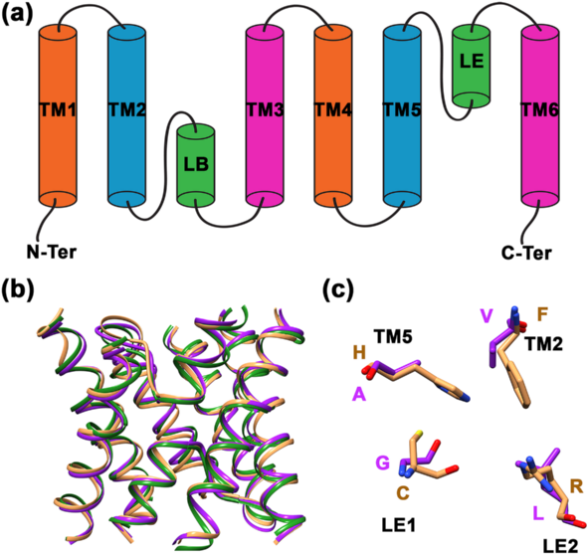
**Topology, structure and selectivity filter of MIP channels. (a)** Topology diagram of a MIP channel exhibiting six transmembrane segments (TM1 to TM6) and the two half-helices formed by the functionally important loops LB and LE. **(b)** Superposition of three MIP channel structures. Water-transporting AQP1 (brown; PDB ID: 1J4N) and glycerol-specific GlpF (green; PDB ID: 1FX8) are superposed on a modeled fungal MIP structure (purple). Only the helical backbone of transmembrane segments and the loop LB and LE are shown for clarity. **(c)** Superposition of residues forming the aromatic/arginine selectivity filter from the water-transporting AQP1 (brown) and a model from a representative example of SIP-like fungal MIP channel (purple). Amino acids in one letter codes are labeled in the respective color of each structure. The contributing transmembrane segments (TM2 or TM5) and the loop positions (LE1 or LE2) are also indicated.

**Figure 2:**
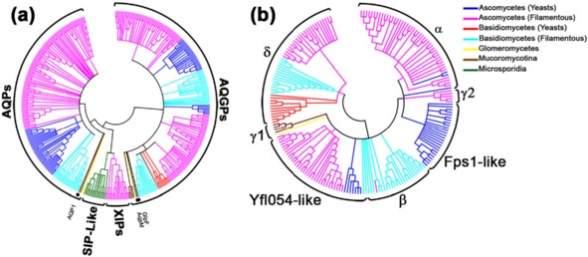
**Phylogenetic analysis of fungal MIP channels. (a)** Phylogenetic analysis of 395 fungal MIP channels. The newly identified “SIP-like” fungal MIP channels are distinct from orthodox AQPs, AQGPs and XIPs. **(b)** Phylogenetic analysis of all fungal AQGPs. The newly identified δ-cluster forms one of the major subfamilies along with α, β, Fps1-like and Yfl054-like AQGPs. γ1 and γ2 form two small clusters. The color of the branches indicates the taxonomy as mentioned in the legends.

Ten MIP sequences representing different subfamilies and organism groups were used as query sequences in the PSI-BLAST and BLAST searches to search the non-redundant database in NCBI (http://www.ncbi.nlm.nih.gov) and UniProt (http://www.uniprot.org) database [52] respectively. Although the MIP sequences are diverse, they all adopt a characteristic hour-glass helical fold consisting of six transmembrane helical segments (TM1 to TM6) and two functionally important loops with half-helices (LB and LE) which meet in the middle of the membrane (Figure 1). In addition to the above sequences, we also used sequences belonging to fungal XIP subfamily [12] as query sequences. Among the 8 phyla present in the kingdom of fungi [53],[54], at the time of database search, genome sequences of 3 phyla were available either in completed form (35 fungal organisms) or in the form of whole genome shotgun sequences (241 fungi). Our search, as described in the Methods section, yielded 326 fungal MIP sequences. Additional 69 sequences were taken from the studies of Xu et al. [51] and many are found in the database of Joint Genome Institute (JGI; http://jgi.doe.gov/). The phylogenetic analysis of all 395 fungal MIP sequences is presented in Figure 2a. It is clearly evident that there are two major clusters representing aquaporins and aquaglyceroporins. The dominant AQP and AQGP clusters contain 163 and 199 members respectively. The third cluster containing the XIP sequences was identified in our earlier studies [12]. In the present study, we report 17 XIP sequences and this group is common to both fungi and plants. This analysis shows another cluster with 16 sequences distinct from the above mentioned three groups. We name this cluster as SIP-like MIP sequences. SIPs form one of the subgroups within plant MIPs [11] and they are small and basic intrinsic proteins. The reasons for naming this fungal MIP cluster as SIP-like sequences are discussed in the following sections. In the case of AQGPs, the subgroups identified in earlier studies [44],[50],[51] are present in our analysis also. We have also identified two additional AQGP subgroups in our analysis (Figure 2b). Sequences, structural models and other associated details of all 395 sequences from 172 different fungal organisms are available in our MIPModDB database (http://bioinfo.iitk.ac.in/MIPModDB) [6]. The accession codes of these sequences from GenBank or JGI are provided in the Additional file 1: Table S1.

### Taxonomical distribution of fungal MIPs

Taxonomical distribution of different clusters and the subgroups are also indicated in Figure 2. The orthodox fungal AQPs are present mainly in the species of Ascomycota (yeast and filamentous) and the rest are found in filamentous Basidiomycota. Only two examples are found in Glomeromycota and a lone representative is from Mucoromycotina. All XIPs are found in Ascomycota filamentous fungi. Among the fungal AQGPs, we have identified two additional subgroups in this study apart from the known subgroups. They have different taxonomic distributions. The phylogenetic tree of all fungal AQGPs is presented in Figure 2b. The Fps1-like AQGPs are found invariably in yeast Ascomycota while the Yfl054-like AQGPs, also known as facultative aquaporins [51], are predominantly identified in yeast and filamentous Ascomycota. The other major subgroups classified as α and β are mainly found in Ascomycota (filamentous) and Basidomycota (filamentous) respectively. There are two small subgroups which we have designated as γ1 and γ2. The γ1 cluster has been previously recognized [51] and is mainly found in the species of Mucoromycotina. The second cluster γ2 is newly recognized in this study and is found in filamentous Ascomycota. The major fungal AQGP subgroup newly identified from this analysis consists of 49 members and the members of this group are found in filamentous Ascomycota and Basidiomycota (yeast & filamentous). We have named this cluster as δ. The new fungal MIP group, which is distinct from orthodox AQPs and AQGPs, are found exclusively in Microsporidia and these parasitic species are known to infect their animal hosts. In this context, it is even more important to understand and characterize this MIP group. We have designated this group as “SIP-like” fungal MIPs since they possess several characteristics of plant SIP subgroup [11] (see below). From this analysis, it appears that some fungal MIP subfamilies such as XIPs and the newly identified SIP-like members are exclusive to a single fungal phylum (Figure 2). However, one has to keep in mind that the present analysis was carried out with limited number of available fungal genome sequences. When more fungal genome sequences are available, it is possible that MIP members from these subfamilies may be found in other fungal phyla also.

### Characterization of new fungal MIP cluster that is distinct from AQPs, AQGPs and XIPs

Sixteen fungal MIPs form a cluster separate from AQPs, AQGPs and XIPs. Average pairwise sequence identity of all these 16 sequences is about 54% and similarity is close to 70%. We also calculated the average pairwise sequence identity between the sequences from the newly identified cluster and all the sequences belonging to the clusters of orthodox AQPs and XIPs. A similar exercise was carried out for all the subgroups of AQGPs also. The results of pairwise sequence analysis are given in Additional file 2: Table S2. Inter-group average sequence identity varies from 16 to 27% and similarity varies from 32 to 44% indicating that this group of sequences exhibits a large degree of divergence from all other fungal MIP sequences.

**Figure 3:**
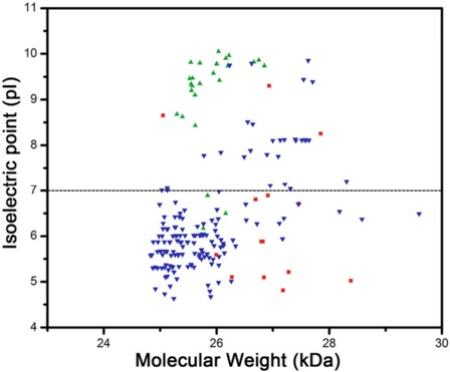
**Relationship between isoelectric point and molecular weight for the newly identified fungal MIP cluster and selected plant MIP subfamilies.** Plot showing isoelectric point versus molecular weight for SIP-like MIPs (red squares), plant TIPs (blue inverted triangles) and plant SIPs (green triangles). One hundred eighty one plant TIPs from 45 species and 25 SIPs from 10 different plant species were considered for this analysis. See also Table 2 for details of the species.

MIP subfamilies within plants have been shown to have different biochemical properties [11]. To find out whether the newly identified fungal MIP group exhibits differential properties, we plotted isoelectric point versus molecular weight for each fungal MIP groups and subgroups. We also carried out comparative studies of fungal MIP groups with plant MIP subfamilies (Figure 3). Our analysis shows that members of the newly identified fungal MIP cluster are smaller in size and have molecular weight similar to that found for the plant TIP and SIP subfamilies. While SIP members are more basic, the new fungal MIP sequences have almost equal distribution of acidic and basic residues.

### Analysis of Ar/R selectivity filter residues

**Figure 4:**
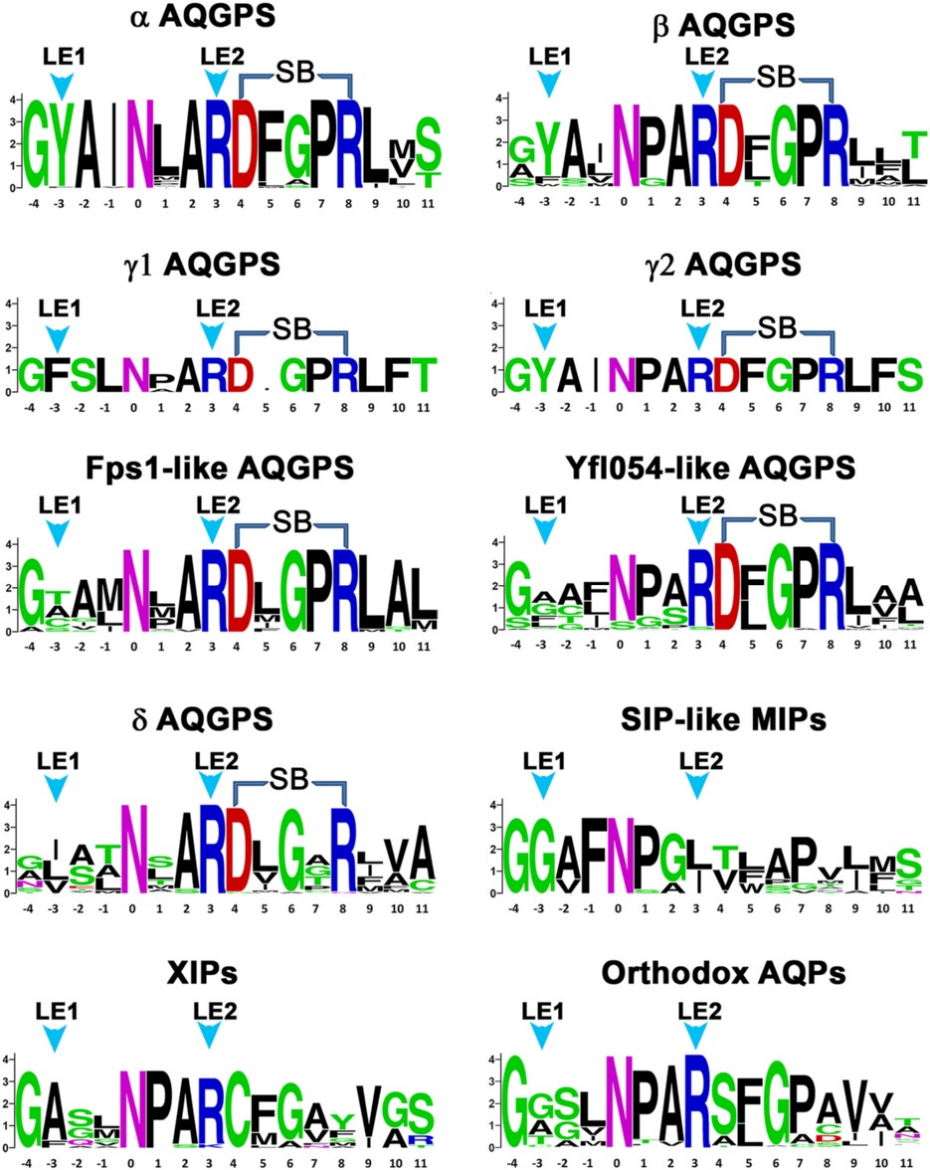
**Sequence logos produced for the loop E region of different fungal MIP groups.** The positions corresponding to the conserved NPA motif are used as the reference point and the position of Asn in the NPA motif is designated as ‘0”. All other amino acids are marked relative to this position. The arrows shown in cyan point to the residues from LE1 and LE2 positions that participate in the formation of the narrow selectivity filter. The acidic and basic residues that can form intra-helical salt-bridge interaction are indicated in the figure. The sequences logos were produced from the web server http://weblogo.berkeley.edu/logo.cgi.

To further characterize the new subfamily, we constructed homology models of all fungal MIP sequences as described in the Methods section. We particularly focused on the aromatic/arginine selectivity filter residues that have been shown to play significant role in determining the selectivity of the solutes to be transported [55]–[57]. The four residues are contributed by TM2, TM5 and the two loop residues from loop E, LE1 and LE2 (Figure 1). As the name suggests, usually the TM2 position is predominantly occupied by an aromatic residue and the LE2 position is almost always an arginine residue. This is true for majority of other groups of fungal MIP sequences except the newly identified family. It has neither aromatic nor arginine residue in its selectivity filter. Both the TM2 and LE2 positions are occupied by bulky hydrophobic residues and small residues (A/S/G) are found in the other two positions TM5 and LE1 (Table 1). Sequence Logo [58] produced for loop E region also clearly highlights this fact (Figure 4). While all the AQGP subfamilies and AQP and XIP clusters possess the highly conserved arginine which forms part of the selectivity filter, only the newly identified fungal MIP cluster is devoid of this arginine residue in its selectivity filter. Since no fungal MIP subgroup has this feature, we looked at the selectivity filter residues of plant SIP and TIP subfamilies. In both cases, we found members in which aromatic or arginine residue is absent in the selectivity filter (Table 2). A significant number of TIPs do not have arginine as part of the selectivity filter. However, there is a histidine in TM2 position which could provide necessary basic character in the place of arginine. Analysis of 25 plant SIPs from different species reveals that none of them have the arginine residue. Although in some cases an aromatic residue is found in TM2/TM5/LE2 position, the selectivity filter of plant SIP members is in general more hydrophobic as in the newly identified fungal MIP group (Table 2).

**Table 1:** Aromatic/arginine selectivity filter residues and substitutions in the conserved NPA motifs in different fungal MIP subgroups

		**Aromatic/arginine selectivity filter**^**c**^				
**Fungal MIP subgroup**^**a**^	**Number of sequences/Total number of sequences**^**b**^	**TM2**	**TM5**	**LE1**	**LE2**	**NPA substitution (%)**^**d**^
FPS-1 like AQGPs	25/26	W	L/I/M/N	T/C/A/S	R	61.7
Facultative	17/42	W	A/S/T/G/D	G/A	R	66.7
(Yfl054-like)	8/42	W	V/I	G	R	
AQGPs	5/42	W	M	L	R	
	8/42	W	G	F/Y	R	
α-cluster AQGPs	46/48	W	G	Y/W/F	R	95.8
β-cluster AQGPs	20/24	W	G	Y	R	12.5
γ1-cluster AQGPs	4/5	T/N/S	C/T	F	R	20
γ2-cluster AQGPs	5/5	W/M/I	G	Y	R	0
δ-cluster AQGPs	49/49	F/Y/M	V/I/A	I/L/V	R	100
AQPs	76/163	F/M/A	H	T/A/C/S	R	
	64/163	F/I/A	E	G	R	28.8
	15/163	Y/F	M/L/V/A	A/G	R	
	8/163	F/M	Q	C/G	R	
XIPs	17/17	N/S/A	S/A/G	A/F	R/K	82.4
SIP-like	16/16	V/I/L/M	A/S/G	G	L/I	81.2

^a^For details of different fungal MIP subgroups, see Figure 1 and the text.

^b^Number of sequences possessing a particular type of Ar/R selectivity filter residues and the total number of sequences belonging to that particular fugal MIP subgroup are given.

^c^The four residues forming the selectivity filter of an MIP channel are contributed by the second (TM2) and fifth (TM5) transmembrane segments and two residues from the loop E region (LE1 and LE2).

^d^Percentage of sequences from that particular fungal MIP subgroup that has substitution(s) in at least one of the conserved NPA motifs.

**Table 2:** Aromatic/arginine selectivity filter residues and substitutions in the conserved NPA motifs in plant subfamilies TIPs and SIPs

		**Aromatic/arginine selectivity filter**^**c**^				
**Plant MIP subfamily**^**a**^	**Number of sequences/Total number of sequences**^**b**^	**TM2**	**TM5**	**LE1**	**LE2**	**NPA substitutions (%)**^**d**^
TIPs^e^	90/181	H	I/V/M	A/G/S	R	1.1
	65/181	H	I/V	A	V/L/I	
	16/181	H/Q/N	T/S/A	A	R	
	5/181	Q	V	A	R	
	3/181	H	V	G	C	
SIPs^f^	13/25	V/F/I/L	V/I	P	N	84.0
	6/25	S/T/Y	H	G	S/A	
	3/25	I/V	V/F	P	I/F	
	3/25	A/S	V	P	N	

^a^For details of different plant MIP subfamilies, see [10]–[12].

^b^Number of sequences possessing a particular type of Ar/R selectivity filter residues and the total number of sequences belonging to TIPs or SIPs are given.

^c^See foot note c of Table 1.

^d^Percentage of sequences from TIPs or SIPs that have substitution(s) in at least one of the conserved NPA motifs.

^e^TIP sequences belong to the following plants:

*Daucus carota*, *Oryza sativa*, *Gossypium hirsutum*, *Kandelia candel*, *Poa pratensis*, *Nicotiana glauca*, *Medicago sativa*, *Brachypodium distachyon*, *Brassica napus*, *Hordeum vulgare*, *Lolium perenne*, *Triticum aestivum*, *Mesembryanthemum crystallinum*, *Wolffia australiana*, *Glycine max*, *Sporobolus stapfianus*, *Solanum lycopersicum*, *Solanum tuberosum*, *Lilium longiflorum*, *Spinacia oleracea*, *Pisum sativum*, *Dendrobium officinale*, *Sorghum bicolor*, *Arabidopsis thaliana*, *Zea mays*, *Cucurbita cv.* Kurokawa Amakuri, *Antirrhinum majus*, *Vitis vinifera*, *Lycopersicon esculentum*, *Raphanus sativus*, *Petroselinum crispum*, *Populus trichocarpa*, *Brassica oleracea*, *Helianthus annuus*, *Posidonia oceanic*, *Pyrus communis*, *Setaria italic*, *Petunia hybrid*, *Medicago truncatula*, *Vernicia fordii*, *Vitis berlandieri x Vitis rupestris*, *Nicotiana tabacum*, *Tulipa gesneriana*, *Lotus japonicas*, *Phaseolus vulgaris.*

^f^Plant SIP sequences have been identified in the following species:

*Brachypodium distachyon*, *Sorghum bicolor*, *Oryza sativa*, *Zea mays*, *Setaria italic*, *Triticum aestivum*, *Arabidopsis thaliana*, *Solanum tuberosum*, *Solanum lycopersicum*, *Populus trichocarpa.*

### Substitutions in the highly conserved NPA boxes

**Figure 5:**
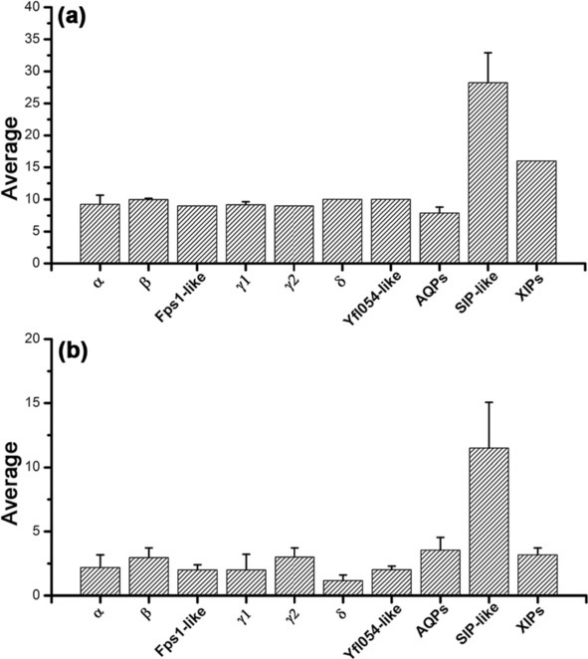
**Analysis of loop D in various fungal MIP groups. (a)** Average length and **(b)** Average number of charged residues of loop D connecting the transmembrane segments TM4 and TM5 are shown for different fungal MIP subfamilies.

The third factor we examined is the substitution in the highly conserved NPA motifs. In majority of the members belonging to the newly identified fungal MIP cluster, alanine residue of NPA motif from loop E has been substituted (Table 1). Substitution of conserved NPA motif is also common in plant SIP subfamily (Table 2). While in almost all plant TIPs both NPA motifs are strictly conserved, substitution in NPA motif of loop B is observed in 21 out of 25 SIP members. Thus the newly identified fungal MIP group resembles more like the plant SIP subfamily in terms of the molecular weight, nature of selectivity filter residues and substitution in the highly conserved NPA motifs. Hence, we have classified this new fungal MIP cluster as “SIP-like” subfamily taking all these factors into account. In our previous studies we have demonstrated that MIP sequences show high group conservation of residues at 17 positions which occur at the helix-helix interface of the hour-glass helical fold [12],[59]. Analysis of the SIP-like members in fungi reveals that 14 out of 17 positions exhibit very high group conservation confirming that the new members have characteristic features of MIP superfamily (Additional file 3: Table S3). Further analysis shows that the SIP-like subfamily members have small N- and C-termini regions and the loop D connecting TM4 and TM5 is the longest in this family compared to all other fungal MIP clusters (Figure 5). This loop in SIP-like MIPs also possesses highest number of charged residues among all fungal MIP subgroups.

### Characterization of δ subfamily of fungal AQGPs

The newly identified SIP-like fungal MIP family is distinct from AQPs, AQGPs and XIPs within fungal MIP family. Within the fungal AQGP group, five different subgroups have already been identified in a recently published study [51]. In the current study, we have also identified two additional subgroups distinct from the previously identified fungal AQGP clusters. The biggest cluster is δ with 49 members (Figure 2b). The average pairwise sequence identity and similarity within this group are about 51% and 67% respectively. This indicates that the sequences within the group are closely related. However, the same cannot be said for AQGP members from other subgroups. The inter-group average sequence identity between δ cluster and other AQGP groups varies from 24% to 30% indicating that δ subgroup members have diverged significantly and are distantly related to other fungal AQGP groups (Additional file 2: Table S2). Hence, our study mainly focused on features that are unique to δ cluster AQGPs and hence, we present our analysis by comparing δ subgroup with other fungal AQGP groups.

**Figure 6:**
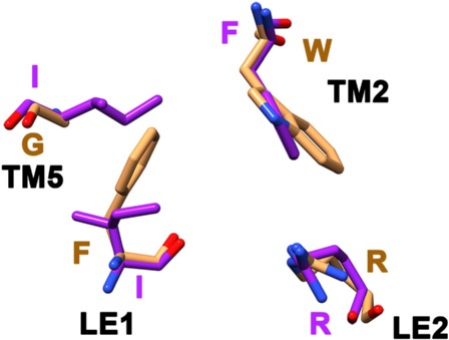
**Aromatic/arginine selectivity filter is novel in δ AQGPs.** Superposition of selectivity filter residues from glycerol-transporting GlpF (brown; PDB ID: 1FX8) and one of the modeled fungal AQGP member from the δ subgroup (purple).

We have analyzed the molecular weight of these sequences as a function of isoelectric point and compared them with other fungal AQGP subgroups. We did not find any feature in this plot that distinguishes δ from other fungal AQGP clusters (Additional file 4: Figure S1). We then analyzed the selectivity filter residues from the homology models we have generated. All the members from this subgroup contain arginine in the LE2 position and the TM2 position is predominantly occupied by an aromatic residue (Table 1). The other two positions from TM5 and LE1 are preferred by bulky aliphatic residues Ile, Leu and Val (Figure 6). This results in a relatively more hydrophobic environment in the selectivity filter region. This also distinguishes δ from α, β, and γ2 members in which two aromatic residues are found in TM2 and LE1 positions and usually a small residue is found in TM5 position (Table 1). In the subgroups Fps1-like and facultative aquaporins, at least one position is occupied by a small residue. Thus it is clear that the δ subgroup MIP members have selectivity filter distinct from other fungal AQGPs. We anticipate that members from this group are likely to transport relatively more hydrophobic solutes.

**Figure 7:**
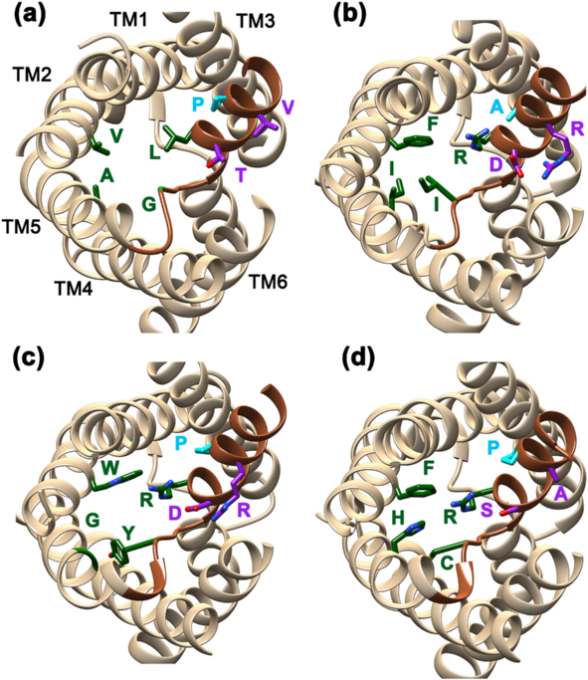
**Intra-helical salt-bridge in the loop E half-helix of fungal AQGPs.** MIP channels from representative members from **(a)** SIP-like, **(b)** δ AQGP, **(c)** α AQGP and **(d)** orthodox AQP clusters. The view is down the channel axis from the extracellular side. The residues forming the selectivity filter are shown in green in stick representation. Loop E is shown in dark brown. Acidic and basic residues forming the intra-helical salt-bridge are shown for the δ and α members. The equivalent positions are shown in SIP-like and orthodox AQP members. With the exception of the δ-subgroup, a proline residue occurs at the intervening positions of the acidic and basic residues and it is highly conserved in all other AQGP subgroups and also in most of the orthodox AQPs. This residue is shown in blue and it is substituted by Ala in the representative δ MIP model shown here.

We have examined the conservation of the signature NPA motifs in loops LB and LE and compared the pattern with other AQGPs. While the NPA motif of loop LB is substituted in some members of δ cluster, the proline residue is invariably replaced in almost every member in the second NPA motif that occurs in LE (Table 1 and Figure 4). This is also observed in α cluster and Fps1-like members. Analysis of sequences in the loop B region reveals that the highly conserved Asn in the NPA motif is substituted by Ser or His in some of δ AQGPs indicating that side-chains of these residues are likely to play a similar role as that of Asn (Additional file 5: Figure S2). Sequence logo of loop LE region reveals strict conservation of an aspartate and an arginine residue near the NPA motif of loop LE in all AQGP members (Figure 4). These two residues are separated by four positions enabling them to form a salt-bridge interaction in the half-helix formed by the LE loop (Figure 7). Such an interaction gives additional stability to the helical region within LE and the residues forming these interactions do not face the channel interior. Such a stabilizing interaction seems to be unique to AQGPs from fungi and other organisms (R. K. Verma, N. D. Prabh and R. Sankararamakrishnan, Unpublished results). The acidic and basic residues are not conserved in orthodox AQPs from fungi, XIPs and the newly identified SIP-like MIPs (Figure 4). Molecular dynamics simulations of mammalian AQP, *E. coli* AQGP and *Plasmodium* AQGP reveal that while the half-helix LE is stable in *E.coli* and *Plasmodium* AQGPs, unwinding of LE half-helix is observed in mammalian AQP [60]. In AQGPs from *E. coli* and *Plasmodium*, the intra-helical salt-bridge interaction gives additional stability to the half-helix in loop LE. Since loop LE contributes two out of four residues (LE1 and LE2) for the Ar/R selectivity filter, we speculate that the unwinding character may help in regulating the channel transport in orthodox AQPs that lack the intra-helical salt-bridge interaction. However in the case of fungal AQGPs, presence of this interaction seems to maintain the helix stable and there could be other mechanisms to regulate the channel function. In this respect, it is important to note the two highly conserved residues, glycine and proline, are present between the acidic and basic residue positions in all fungal AQGP groups except the δ cluster of AQGPs (Figure 4). Both these residues are known to be helix breakers. It appears that the helix stability of loop E half-helix is balanced by two opposing forces, one with stabilizing interaction and the other with the tendency to break helices. In the case of δ cluster, the proline residue is not conserved (Figure 4). Hence, we can anticipate that the half-helix in loop E in δ cluster will be relatively more stable than other fungal AQGPs.

### γ2 cluster of AQGPs

This small group contains only 5 members forming a separate cluster within the fungal AQGPs (Figure 2b) and all of them belong to filamentous Ascomycota. Their selectivity filter contains a Tyr residue at LE1 position which seems to be a feature shared by many members of α and β clusters (Table 1). The TM2 position is preferred by a bulky residue (Trp/Met/Ile) and LE2 position is occupied by Arg. There is no strikingly different feature observed in this small group. Perhaps, a larger dataset would help to identify unique characteristics of small clusters like γ1 and γ2.

## Discussion

### Fungal MIPs are as diverse as plant MIPs

The diversity of plant MIPs has been established in many earlier studies [10]–[12]. Only few studies identified and addressed the extent of diversity in fungal MIPs [44],[50],[51]. In the present study, by identifying two major clusters and one minor cluster within the fungal MIP superfamily, we have determined that fungal MIPs are as diverse as their plant counterparts. Till date, the only MIP subgroup common to both plant and fungal species has been found to be XIPs [12]. However, by systematically searching the genome sequences of fungal species, we have recognized another group in fungal MIPs which has common features with plant SIP subfamily. Although phylogenetic analysis cluster the newly identified “SIP-like” fungal MIPs separately from the plant SIPs, several features are shared by the two MIP families from the two different species group. The small size of the channel protein, the nature of selectivity filter residues and substitutions in the conserved NPA boxes indicate that the new fungal MIP cluster shares some characteristic features with plant SIPs.

### Unique selectivity filter suggests that SIP-like MIPs are likely to transport larger hydrophobic solutes

Very few functional studies have been carried on fungal MIPs [39],[41],[44],[45]. Experimental, structural and simulation studies have emphasized the role of the aromatic/arginine selectivity filter in MIP channel’s transport and selectivity [55]–[57],[61]–[64]. In this context, it would be interesting to at least make a speculation regarding the transport properties of SIP-like fungal MIPs based on the selectivity filter residues. Even for plant SIPs, not many studies have been reported regarding their function. When *Arabidopsis* SIPs (AtSIPs) were expressed in yeast, at least one of the two members displayed water channel activity [65]. This water transport has been reported in spite of the fact that the aromatic/arginine selectivity filter of AtSIPs lacks the arginine residue. In a recent study [34], an aquaporin AQP2 from the protozoan parasite *Trypanosoma brucei gambiense* or *Trypanosoma brucei rhodesiense* has been shown to have an unusual selectivity filter comprising of large hydrophobic residues IVLL. *T. brucei* causes sleeping sickness in humans and with all hydrophobic residues in the selectivity filter without any aromatic and arginine residue, this aquaporin has been shown to play a vital role in increased cross resistance to the drugs melarsoprol and pentamidine [66]. Based on their studies, Baker *et al*. [34] have suggested that *T. brucei* aquaporin AQP2 may function as a transporter of pentamidine and melarsoprol drugs. Both drugs with molecular mass above 330 Da are larger molecules compared to glycerol (92 Da). It is suggested that the unique selectivity filter of AQP2 from *T. Brucei* with bulky hydrophobic residues may facilitate the transport of large molecules.

We have also examined all non-plant MIPs whose selectivity filter lacks arginine residue. In MIPModDB [6] which has more than 1000 MIPs, we could find only 52 examples in which arginine is absent in the selectivity filter. Among them, only 17 of them including mammalian AQP12 have features similar to that found in SIP-like fungal MIPs (two bulky hydrophobic residues and two small residues in the selectivity filter). We found that in mammalian AQP12, both TM2 and LE2 positions are occupied by Leu residues and Ala is found in TM5 and LE1 positions. Although no direct functional studies are reported for AQP12, it has been speculated that AQP12 may regulate proper secretion of pancreatic fluid or digestive enzymes [67],[68]. Thus, plant SIPs, AQP12 and AQP2 of *T. Brucei* also have selectivity filters similar to fungal SIP-like MIPs with all four positions occupied by hydrophobic residues and arginine is absent. Based on the available literature on other MIPs with similar features, we conclude that the SIP-like fungal MIPs are likely to transport solutes which are novel, hydrophobic and larger in size. The same argument may hold good for the plant SIPs also.

### Loop D in SIP-like members may play a role in regulating the channel function

The SIP-like fungal MIPs exhibit some characteristic features in loop D which connects the TM4 and TM5 helices. The average length of loop D is the longest in SIP-like cluster compared to all other fungal MIP subgroups (Figure 5). Moreover, large number of charged residues is found in loop D in SIP-like members. This observation indicates that loop D in SIP-like MIPs can play a special role in the function of these channels. Loop D in some mammalian and plant aquaporin channels have been shown to be important in channel gating and regulation of channel function. Experimental and computational studies in AQP1, spinach aquaporin SoPIP2;1 and AQP4 have suggested a role for loop D in channel gating [69],[70] or binding site for channel agonists [71] or antagonists [72]. Specific residues in loop D which respond to pH have been shown to be responsible for the closed state in AQP1 channel [73]. The arginine-rich loop D in AQP1 has also been shown to bind cGMP which seems to initiate channel opening [74]. Loop D in AQP4 serves as a possible region for metal-binding activity and has also been implicated in regulating the channel activity [75],[76]. Structural studies on spinach aquaporin characterized the open and closed states and exhibited two different conformations of loop D revealing that the gating mechanism at molecular level involves a large movement of this loop [77]. The available experimental and simulation studies provide a clue for the role of loop D in fungal SIP-like MIPs. The charged residues in this loop could be involved in binding ions or molecules that could trigger the channel opening and closing. The longer loop in this subfamily can easily adopt different conformations that can regulate the channel transport. Both possibilities can also simultaneously exist implying that the loop D in SIP-like channels should be given specific attention while investigating the structure-function relationship of these subgroup members.

### SIP-like channels could be attractive targets for some anti-fungal diseases

It is interesting to note that all the SIP-like members are from the lower fungi Microsporidia. We did not find even a single example from higher fungi. Although Micorsporidia were earlier classified as part of the kingdom protozoa, these spore-forming unicellular parasites are now known to be fungi. They infect both humans and animals and cause microsporidiosis in humans [78]. As human pathogens they cause diarrhea and infections in immune-compromised individuals (Additional file 6: Table S4). Hence, understanding the host-pathogen interactions and the infection mechanism is extremely important that will aid in developing anti-fungal drugs. SIP-like members in Microsporidia are unique in several respects. They are small in size and the channels formed by them have hydrophobic selectivity filters. The loop D in these channels is longer and is highly charged. The nature of molecules that are transported through these channels and the role of loop D in regulating the channel function have to be first experimentally investigated. Although human AQP12 has similar selectivity filter, phylogenetic analysis clearly clusters them into separate clades. Hence, SIP-like MIP channels in disease causing Microsporidial fungi can be considered as attractive drug targets.

### Fungal AQGPs have the largest number of subfamilies

With the identification of δ and γ2 AQGP subfamilies in this study, fungal AQGPs can be classified into seven different subgroups (Fps1-like, facultative, α, β, γ1, γ2 and δ). To our knowledge, the kingdom of fungi possesses the largest subfamilies within the major group of AQGPs. This makes the fungal AQGPs as the most diverse among all known AQGPs compared to any major organism groups. In majority of fungal AQGPs, Trp is found to be preferred in the TM2 position of the selectivity filter and Arg is absolutely conserved in LE2 position of all fungal AQGPs (Table 1). Most of the variations are seen in TM5 and LE1 positions. Members of γ1 AQGPs and the newly identified δ subgroup of AQGPs defy the trend observed in TM2 position. While Trp is absent in members of both the clusters, a small polar residue (Thr, Ser or Asn) is found in γ1 at TM2 position. However in δ subgroup, bulky residues of hydrophobic nature (Phe, Tyr, Met, Val, Ile and Leu) are found in TM2, TM5 and LE1 positions. As in SIP-like MIPs, the selectivity filter of δ AQGPs appears to be unique. We searched MIPModDB database with more than 1000 MIP members looking for examples with similar features in their selectivity filters. We could find only four MIPs in which the channel selectivity filter is formed by three bulky hydrophobic residues and the fourth position is occupied by an arginine residue.

As mentioned earlier, δ subgroup of AQGPs has been found in filamentous Ascomycota and yeast and filamentous Basidiomycota. Many examples from this species group are known to be plant pathogens [79] (Additional file 7: Table S5). Members belonging to this group have been identified to cause serious diseases in wide range of plants including agriculturally important cereals such as rice, wheat, rye and barley. They also affect economically important crops like cotton. These pathogens display resistance to fungicides and δ subgroup of AQGPs can be considered as an important target to contain these plant pathogens.

### Loop E as a marker to study the evolution of MIPs

Loop E provides two residues for the selectivity filter. It also possesses one of the two conserved NPA motifs. While the other conserved NPA motif resides in loop B, majority of the positions in this loop show variations (Additional file 5: Figure S2). In the case of loop E, this region seems to show some distinctive features depending upon the subgroups. Six out of seven AQGPs exhibit a conserved motif RDxGPR next to the NPA motif in loop E (Figure 4). A very high conservation of acidic and basic residues separated by four positions will enable the formation of salt-bridge interaction in the half-helix. However, as mentioned earlier, the presence of two helix destabilizing residues Gly and Pro will weaken the stability of the same helix. In δ AQGPs, the motif present is RDxGxR and the Pro residue next to Gly is mostly substituted by other residues. Hence, we can think of the half-helix in δ AQGPs as relatively more stable compared to all other fungal AQGP subgroups. In XIPs, the acidic and basic residues are replaced by a strictly conserved cysteine and mostly bulky residues (Tyr, Phe or Met) respectively. The motif in XIPs is found to be RCx[G/A]xx. SIP-like members exhibit no such conserved motif in this region. In orthodox AQPs, the acidic and basic residues are replaced by small residues and the conservation pattern in the same region is R[S/A]xG[P/A][A/C/D/S]. It should be noted that the absence of intra-helical salt-bridge and the conservation of Gly and to some extent Pro in AQPs will render this half-helix relatively less stable compared to the same region in AQGPs in general and δ-AQGPs in particular. An extensive bioinformatics analysis of MIPs from microbial organisms to mammals in loop E is necessary and this may spring some surprising insights into the evolution of MIPs.

## Conclusions

The role of different fungal proteins in the symbiotic relationship with plants needs to be clearly established. Similarly, host-pathogen interactions at the time of fungi-induced infection require understanding at the molecular level. In this context, understanding the evolution, function and diversity of fungal MIP channels has become very significant. Experimental studies clearly reveal that MIP channels are important players in plant-fungi interactions. Identification of new targets for anti-fungal drugs is a major goal for several human diseases caused by fungi and MIP channels could be considered as attractive anti-fungal targets. In the present study, analysis of fungal genome sequences has identified additional MIP channels in different species groups within the kingdom of fungi. Phylogenetic analysis of nearly 400 fungal MIP channels has revealed the existence of a new MIP cluster completely distinct from the orthodox AQP and AQGP channels. Further sequence analysis and homology modeling studies indicate features that are shared between the new fungal MIP cluster and the plant SIP subfamily. The size of the protein, chemical nature of the residues that form the narrow aromatic/arginine selectivity filter and the substitutions found in the conserved NPA motifs are the common characteristics between the new family and the plant SIP channels. Hence in addition to the XIPs, this “SIP-like” fungal MIP channels can possibly be another evolutionary link between the plants and fungi. Since SIP-like channels are observed only in Microsporidia which are the unicellular parasites, they can also be considered as drug targets for developing anti-fungal drugs in human infections caused by fungi.

We have also identified one major subgroup and another minor cluster within the fungal AQGP family. The δ-subgroup with 49 members have unique selectivity filter very rarely found in other MIP channels. With the discovery of these two new AQGP subfamilies, it appears that fungal AQGPs are the most diverse among all known AQGPs. Many fungal species possessing δ-AQGPs also are known to act as plant pathogens. The newly identified δ-group MIP channels can be exploited to contain some of the serious infections affecting agriculturally and economically important crops.

With very few functional studies available in fungal MIP channels, the present study has given a picture of the diverse fungal organisms evolved with two new MIP subfamilies with unique selectivity filter residues. The possible solutes that are transported through these narrow regions within the channel interior, the role of loop D in SIP-like channels and the importance of these channels in the fungal life cycles are some of the immediate questions that have to be addressed by researchers in this field.

## Methods

### Identification of fungal MIP sequences

The non-redundant database of NCBI (http://www.ncbi.nlm.nih.gov) and the UniProtKB (http://www.uniprot.org) database [52] were searched using known AQP and AQGP sequences as query. The UniProt accession IDs of these sequences are P0AER0, P47865, P60844, Q6J8I9, Q9C4Z5, Q41372, Q8WPZ6, P55064, P55087 and F2QVG4. These sequences belong to different species and organism groups and they come from bacteria (*Escherichia coli*), mammals (*Bos taurus*, *Ovis aries and Homo sapiens*), archaea (*Methanothermobacter marburgensis*), plant (*Spinacia oleracea*), protozoan parasite (*Plasmodium falciparum*) and yeast (*Komagataella pastoris*). The three-dimensional structures of all these channels have been determined. In addition to these sequences, we have also considered the sequences belonging to the XIP subfamily from fungal species as query sequences previously identified in our laboratory [12]. We used PSI-BLAST [80] on NCBI non-redundant database to identify fungal MIP sequences. For each sequence, three iterations were performed with threshold e-value 0.001. When going from previous iteration to the next iteration, we included only those hits whose expect value was 0.001 or less to generate the position-specific scoring matrix that can be used in the next iteration. This is to make sure that the PSSM is not corrupted and at the same time diverse MIP sequences can be obtained as hits. BLASTp was used with UniProt database with the same query sequences. Additionally, we also used tBLASTn against NCBI GenBank database to retrieve additional fungal MIP sequences. The newly identified sequences from PSI-BLAST and tBLASTn searches were again used as query sequences and second round of search was carried out using the same tools. This exercise was repeated until no new MIP sequences were found.

We then used the program CD-HIT [81],[82] on the set all fungal MIP sequences thus obtained to remove any redundancy. It is possible that some of the MIP sequences in this non-redundant set may be partial or may not have all the features associated with a MIP channel protein. In order to find out the partial sequences or sequences that don’t have all MIP features, we adopted the following strategy. We used a multiple sequence alignment program PRALINE (http://www.ibi.vu.nl/programs/pralinewww/) to align all the fungal MIP sequences [83]. There are advantages using PRALINE. It uses profile-based approach to align sequences that have low sequence identity. It also guides the sequence alignment using secondary structure information by employing different scoring matrices for helix, strand or coil. For each MIP sequence, we used TMHMM [84] and PSIPRED [85] which are available as part of the PRALINE toolkit to predict transmembrane regions and secondary structures respectively. The multiple sequence alignment thus created was examined to find out the following features specific to MIP family sequences. These are (a) presence of two NPA or NPA-like motifs, (b) presence of six transmembrane segments and two functionally important loops possessing the features characteristically present in MIP channels and (c) group-based conservation of small and weakly polar residues in most of the 17 positions that typically occur in the helix-helix interface of MIP hour-glass helical fold [59]. Only those sequences which satisfy all the three conditions were considered further for analysis.

We identified 326 fungal MIP sequences from the database search described above. We also included additional 69 fungal MIP sequences from the recently published work of Xu et al. [51] which fulfilled all our criteria. In total, 395 fungal MIP sequences were considered for phylogenetic analysis.

### Phylogenetic analysis

To understand the fungal MIP diversity, the evolution of MIP family and to enable comparison with other groups of species, we performed phylogenetic analysis on all fungal MIP sequences using MEGA 5.0 suite of software [86]. Two different clustering algorithms, namely neighbor-joining and maximum parsimony methods, were used to derive a phylogenetic tree. Reliability of individual branches of the tree was estimated by performing bootstrapping with 1000 replicates. We applied 50% majority rule so that branches with less than 50% confidence level were collapsed to get the final tree topology. The fungal MIP subgroups obtained from this tree was considered for further analysis. The groupings obtained from this tree were validated by comparing the trees obtained from the two different clustering methods. We also used different multiple sequence alignments as input for MEGA 5.0 obtained from various MSA tools like Clustal-W [87], Clustal-Ω [88] and MUSCLE [89] and the resulting trees generated from the newly obtained multiple sequence alignments were compared.

### Homology modeling of fungal MIP sequences

Three-dimensional structures of all fungal MIP sequences were modeled using the protocol developed in our laboratory earlier [12],[59]. Briefly, the software MODELLER version 9.10 [90],[91] was used to build the structure and the experimentally determined high-resolution MIP structures from mammalian AQP1 (PDB ID:1J4N) [92], bacterial GlpF (PDB ID: 1FX8) [93] and archael AQPM (PDB ID: 2F2B) [94] were used as templates. The target-template sequence alignment was manually examined to find out whether there are gaps in the transmembrane helical regions or in the functionally important loop regions which possess the highly conserved NPA motifs. We also looked at the high conservation of at least one residue in each transmembrane helical segment (see above). Among the ten models built for each fungal MIP sequence, the one with the optimum MODELLER objective function was selected. Loops and side-chain conformations of non-conserved residues were further refined using the MODELLER’s loop optimization protocol and the program SCWRL3 [95] respectively. The resultant model was minimized using GROMACS ver. 4.5 [96] and the quality of the model was examined using PROCHECK [97].

No human or animal experiments were carried out in this study.

## Abbreviations

MIPs: Major intrinsic proteins

AQPs: Aquaporins

AQGPs: Aquaglyceroporins

XIPs: X-intrinsic proteins

SIPs: Small and basic intrinsic proteins

TIPs: Tonoplast intrinsic proteins

## Competing interests

The authors declare that they have no competing interests.

## Authors’ contributions

RS conceived the study. RKV carried out the sequence search, phylogeneic analysis and homology modeling on fungal channels. NDP and RKV performed the studies on plant channels. RKV, NDP and RS analyzed the results. RS wrote the manuscript. All authors read and approved the final manuscript.

## Additional files

## Supplementary Material

Additional file 1: Table S1. Contains accession IDs of all fungal MIPs belonging to different subgroups. Click here for file

Additional file 2: Table S2. Contains inter- and intra-group average pairwise sequence identities and similarities of all fungal MIP families. Click here for file

Additional file 3: Table S3. Shows group conservation of small and weakly polar residues at the helix-helix interface calculated for all fungal MIP subgroups. Click here for file

Additional file 6: Table S4. Contains list of pathogenic fungi that have at least one SIP-like MIP channel. Click here for file

Additional file 7: Table S5. Contains list of plant pathogenic fungi that have at least one member from the δ subgroup of AQGPs. Click here for file

Additional file 4: Figure S1. Relationship between isoelectric point and molecular weight for all the fungal AQGP groups. Click here for file

Additional file 5: Figure S2. Contains sequence logos produced for Loop B region for different fungal MIP groups. Click here for file
