# Supplementary material for: New subfamilies of major intrinsic proteins in fungi suggest novel transport properties in fungal channels: implications for the host-fungal interactions
Source: BMC Evol Biol. 2014 Aug 12;14:173. doi: 10.1186/s12862-014-0173-4 (PMC4236510; doi:10.1186/s12862-014-0173-4)
Supplement: Additional file 1: Table S1. — Contains accession IDs of all fungal MIPs belonging to different subgroups. [file s12862-014-0173-4-S1.doc]

**able S1: Genbank or JGI accession codes for fungal MIPs belonging to different subgroups**

| **α AQGPs** | | | |
| --- | --- | --- | --- |
| **S. NO.** | **MIPModDB ID** | **GENBANK ACCESSION** | **JGI PROTEIN ID** |
| 1 | AJDERM1353 | EGE78852.1 | - |
| 2 | AJDERM0905 | EEQ71497.1 | - |
| 3 | AJDERM1323 | EEQ90809.1 | - |
| 4 | PABRAS0898 | EEH49996.1 | - |
| 5 | PABRAS0901 | EEH34869.1 | - |
| 6 | AJCAPS0911 | EEH03543.1 | - |
| 7 | AJCAPS1348 | EGC47864.1 | - |
| 8 | UNREES0904 | EEP78744.1 | - |
| 9 | COIMMI1314 | XP_001239082.1 | - |
| 10 | COPOSA0910 | EER29911.1 | - |
| 11 | COPOSA1337 | EFW18977.1 | - |
| 12 | TRREES1370 | EGR45687.1 | - |
| 13 | TRVIRE1536 | - | 184650 |
| 14 | ZYTRIT1415 | XP_003853787.1 | - |
| 15 | BOFUCK1554 | EMR90282.1 | - |
| 16 | GLLOZO1491 | EHK98241 | - |
| 17 | MICANI0893 | EEQ34931.1 | - |
| 18 | ARGYPS1331 | XP_003172410.1 | - |
| 19 | TRTONS1350 | EGD95973.1 | - |
| 20 | TRRUBR1352 | XP_003237178.1 | - |
| 21 | MYFIJI1518 | - | 133800 |
| 22 | GRCLAV1033 | EFX02739.1 | - |
| 23 | ASNIDU1049 | CBF75100.1 | - |
| 24 | ASFUMI0307 | XP_750737.1 | - |
| 25 | NEFISC1315 | XP_001257964.1 | - |
| 26 | ASCLAV0903 | EAW06528.1 | - |
| 27 | PECHRY1046 | XP_002565260.1 | - |
| 28 | ASFLAV0900 | EED48588.1 | - |
| 29 | ASORYZ1040 | BAE57020.1 | - |
| 30 | MAGRIS0851 | EDJ99905.1 | - |
| 31 | MAORYZ1407 | XP_003717502.1 | - |
| 32 | VEALBO0912 | EEY19696.1 | - |
| 33 | VEDAHL1391 | EGY16575.1 | - |
| 34 | NEHAEM0909 | EEU37218.1 | - |
| 35 | FUOXYS1381 | EGU81740.1 | - |
| 36 | GLGRAM1020 | EFQ34814.1 | - |
| 37 | TRREES1374 | EGR50515.1 | - |
| 38 | TRVIRE1537 | - | 196947 |
| 39 | COMILI1389 | EGX97077.1 | - |
| 40 | MEACRI1037 | EFY86927.1 | - |
| 41 | MEANIS1340 | EFZ01884.1 | - |
| 42 | YALIPO0484 | XP_504820.1 | - |
| 43 | YALIPO0485 | XP_503595.1 | - |
| 44 | ZYTRIT1414 | XP_003853380.1 | - |
| 45 | CLGRAY1482 | - | 115933 |
| 46 | LEMACU1026 | CBX94430.1 | - |
| 47 | ZYTRIT1412 | XP_003850801.1 | - |
| 48 | PIPAST0864 | XP_002494229.1 | - |

| **β AQGPs** | | | |
| --- | --- | --- | --- |
| **S. NO.** | **MIPModDB ID** | **GENBANK ACCESSION** | **JGI PROTEIN ID** |
| 1 | LABICO0914 | EDR10355.1 | - |
| 2 | LABICO1509 | JQ585597 | - |
| 3 | LABICO0918 | EDR10356.1 | - |
| 4 | LABICO1508 | JQ585596 | - |
| 5 | LABICO0921 | EDR10549.1 | - |
| 6 | LABICO1506 | JQ585594 | - |
| 7 | COCINE0915 | EAU83999.1 | - |
| 8 | LABICO1507 | JQ585595 | - |
| 9 | LABICO0916 | EDR04633.1 | - |
| 10 | LABICO1512 | - | 443240 |
| 11 | SCCOMM1010 | XP_003032759.1 | - |
| 12 | PHCHRY1521 | - | 5154 |
| 13 | WOCOCO1544 | - | 23700 |
| 14 | POPLAC1526 | - | 114217 |
| 15 | PHCHRY1522 | - | 5155 |
| 16 | TRVERS1532 | - | 121294 |
| 17 | SELACR1366 | EGN98476.1 | - |
| 18 | PUGRAM1553 | EFP84756.2 | - |
| 19 | MELARI1356 | EGG04320.1 | - |
| 20 | MIOSMU1498 | GAA93674 | - |
| 21 | SPREIL1480 | CBQ72167 | - |
| 22 | USMAYD0865 | EAK82563.1 | - |
| 23 | SPREIL1479 | CBQ71719 | - |
| 24 | GLGRAM1017 | EFQ30640.1 | - |

| **γ1 AQGPs** | | | |
| --- | --- | --- | --- |
| **S. NO.** | **MIPModDB ID** | **GENBANK ACCESSION** | **JGI PROTEIN ID** |
| 1 | GLINTR1504 | JQ412060 | - |
| 2 | PHBLAK1523 | - | 141336 |
| 3 | PHBLAK1525 | - | 60581 |
| 4 | PHBLAK1524 | - | 143660 |
| 5 | MUCIRC1516 | - | 185371 |

| **γ2 AQGPs** | | | |
| --- | --- | --- | --- |
| **S. NO.** | **MIPModDB ID** | **GENBANK ACCESSION** | **JGI PROTEIN ID** |
| 1 | ASNIDU0884 | XP_658434.1 | - |
| 2 | ASFLAV0938 | EED45967.1 | - |
| 3 | PECHRY1550 | XP002560691 | - |
| 4 | ASNIGE1317 | XP_001400456.1 | - |
| 5 | ASKAWA1496 | GAA84320 | - |

| **Fps1-like AQGPs** | | | |
| --- | --- | --- | --- |
| **S. NO.** | **MIPModDB ID** | **GENBANK ACCESSION** | **JGI PROTEIN ID** |
| 1 | ASGOSS0305 | NP_983336.1 | - |
| 2 | ASGOSS1329 | NP_983336.2 | - |
| 3 | ERCYMB1395 | XP_003648500.1 | - |
| 4 | LATHER0843 | XP_002553728.1 | - |
| 5 | KLWALT1511 | Kwal20572 | - |
| 6 | TEBLAT1425 | XP_004180714.1 | - |
| 7 | NACAST1400 | XP_003675943.1 | - |
| 8 | KAAFRI1422 | XP_003959763.1 | - |
| 9 | KAAFRI1423 | XP_003959772.1 | - |
| 10 | KLLACT0412 | XP_453974.1 | - |
| 11 | KLMARX1474 | AAS47031 | - |
| 12 | CAGLAB0342 | XP_445856.1 | - |
| 13 | VAPOLY0862 | EDO14958.1 | - |
| 14 | KAAFRI1421 | XP_003959066.1 | - |
| 15 | NADAIR1398 | XP_003671440.1 | - |
| 16 | TEBLAT1426 | XP_004180716.1 | - |
| 17 | ZYROUX0847 | XP_002498330.1 | - |
| 18 | TODELB1403 | XP_003682759.1 | - |
| 19 | CAGLAB0343 | XP_445318.1 | - |
| 20 | NADAIR1397 | XP_003670053.1 | - |
| 21 | NACAST1399 | XP_003673249.1 | - |
| 22 | SACERE1318 | EDN59506.1 | - |
| 23 | SACERE1325 | EEU06572.1 | - |
| 24 | SACERE1477 | CAA38096 | - |
| 25 | SACERE0481 | NP_013057.1 | - |
| 26 | SACERE1345 | EGA77792.1 | - |

| **Yfl054-like AQGPs (Facultative AQPs)** | | | |
| --- | --- | --- | --- |
| **S. NO.** | **MIPModDB ID** | **GENBANK ACCESSION** | **JGI PROTEIN ID** |
| 1 | SACERE0480 | NP_116601.1 | - |
| 2 | SACERE1495 | GAA23030 | - |
| 3 | SACERE1322 | EDV12566.1 | - |
| 4 | LATHER0848 | XP_002551817.1 | - |
| 5 | KLWALT1510 | Kwal15269 | - |
| 6 | TODELB1401 | XP_003680120.1 | - |
| 7 | SAPOMB0854 | NP_592788.1 | - |
| 8 | WASEBI1539 | - | 32912 |
| 9 | WASEBI1540 | - | 59835 |
| 10 | ASCLAV0906 | EAW12203.1 | - |
| 11 | NEFISC0899 | EAW17077.1 | - |
| 12 | ASNIDU0842 | XP_680887.1 | - |
| 13 | ASFLAV0908 | EED52139.1 | - |
| 14 | ASORYZ1030 | XP_001825721.2 | - |
| 15 | TRVIRE1534 | - | 41159 |
| 16 | ASNIGE1476 | - | 201277 |
| 17 | ASNIGE1336 | XP_001397337.2 | - |
| 18 | ASNIGE1042 | CAK42703.1 | - |
| 19 | TASTIP0902 | EED24013.1 | - |
| 20 | ASTERR0311 | XP_001217233.1 | - |
| 21 | SCSCLE0896 | EDO00948.1 | - |
| 22 | BOFUCK1320 | XP_001553449.1 | - |
| 23 | MYFIJI1517 | - | 35307 |
| 24 | PYTRIT0913 | EDU44452.1 | - |
| 25 | PYTERE1363 | XP_003298283.1 | - |
| 26 | COHETE1486 | - | 1030135 |
| 27 | PHNODO0856 | EAT77624.2 | - |
| 28 | LEMACU1025 | CBX92322.1 | - |
| 29 | GIZEAE0859 | XP_383956.1 | - |
| 30 | FUOXYS1382 | EGU87897.1 | - |
| 31 | ZYTRIT1411 | XP_003848321.1 | - |
| 32 | ASFLAV0894 | EED51102.1 | - |
| 33 | ASORYZ1547 | XP001821574 | - |
| 34 | SCSCLE0892 | EDN99912.1 | - |
| 35 | PECHRY1047 | XP_002566055.1 | - |
| 36 | ASTERR0310 | XP_001218366.1 | - |
| 37 | ASNIGE1041 | CAK41634.1 | - |
| 38 | ASNIGE1334 | XP_001396373.2 | - |
| 39 | ASKAWA1497 | GAA86900 | - |
| 40 | ASCLAV0897 | EAW09088.1 | - |
| 41 | NEFISC0907 | EAW25680.1 | - |
| 42 | ASFUMI0841 | EDP47128.1 | - |

| **δ AQGPs** | | | |
| --- | --- | --- | --- |
| **S. NO.** | **MIPModDB ID** | **GENBANK ACCESSION** | **JGI PROTEIN ID** |
| 1 | PSFIJI1456 | EME81145.1 | - |
| 2 | DOSEPT1457 | EME42280.1 | - |
| 3 | XAPARI1546 | - | 68791 |
| 4 | EXDERM1455 | EHY52891.1 | - |
| 5 | SPMUSI1454 | EMF12659.1 | - |
| 6 | THTERR1404 | XP_003652729.1 | - |
| 7 | ZYTRIT1413 | XP_003853006.1 | - |
| 8 | MABRUN1458 | EKD14690.1 | - |
| 9 | SCSCLE1321 | XP_001596187.1 | - |
| 10 | BOFUCK0939 | EDN25813.1 | - |
| 11 | BOFUCK1453 | CCD54391.1 | - |
| 12 | PEMARN0922 | EEA24821.1 | - |
| 13 | TASTIP0924 | EED18600.1 | - |
| 14 | COMILI1387 | EGX93816.1 | - |
| 15 | BEBASS1459 | EJP62256.1 | - |
| 16 | TRATRO1449 | EHK47676.1 | - |
| 17 | TRVIRE1448 | EHK17234.1 | - |
| 18 | TRREES1371 | EGR46161.1 | - |
| 19 | TADEFO1452 | CCG81275.1 | - |
| 20 | VEALBO0930 | EEY23678.1 | - |
| 21 | VEDAHL1393 | EGY19188.1 | - |
| 22 | GAGRAM1450 | EJT74755.1 | - |
| 23 | MAORYZ1406 | XP_003711708.1 | - |
| 24 | MAORYZ1451 | ELQ43205.1 | - |
| 25 | MELARI1354 | EGF98244.1 | - |
| 26 | TRASAH1445 | EJT48151.1 | - |
| 27 | SPREIL1443 | CBQ72724.1 | - |
| 28 | PSHUBE1442 | GAC94525.1 | - |
| 29 | USHORD1441 | CCF52279.1 | - |
| 30 | PSANTA1444 | GAC72099.1 | - |
| 31 | USMAYD1431 | XP_758316.1 | - |
| 32 | MIOSMU1446 | GAA93901.1 | - |
| 33 | SPROSE1528 | - | 24618 |
| 34 | RHGLUT1376 | EGU12015.1 | - |
| 35 | RHTORU1447 | EMS24130.1 | - |
| 36 | LABICO0950 | EDR03473.1 | - |
| 37 | LABICO1438 | AFJ15556.1 | - |
| 38 | LABICO1514 | - | 482072 |
| 39 | PUSTRI1439 | EIN03700.1 | - |
| 40 | FOMEDI1434 | EJD06583.1 | - |
| 41 | STHIRS1440 | EIM91284.1 | - |
| 42 | CESUBV1436 | EMD37593.1 | - |
| 43 | TRVERS1435 | EIW63166.1 | - |
| 44 | TRVERS1531 | - | 113714 |
| 45 | CESUBV1433 | EMD39794.1 | - |
| 46 | CESUBV1437 | EMD36601.1 | - |
| 47 | WOCOCO1543 | - | 121322 |
| 48 | FIRADI1432 | CCM00974.1 | - |
| 49 | POPLAC1527 | - | 127849 |

| **SIP-like Fungal MIPs** | | | |
| --- | --- | --- | --- |
| **S. NO.** | **MIPModDB ID** | **GENBANK ACCESSION** | **JGI PROTEIN ID** |
| 1 | ENCUNI0391 | NP_586002.1 | - |
| 2 | ENCUNI1470 | AGE95845.1 | - |
| 3 | ENROMA1465 | AFN83320.1 | - |
| 4 | ENINTE1462 | XP_003073192.1 | - |
| 5 | ENHELL1419 | XP_003887579.1 | - |
| 6 | ENHELL1460 | Q1M1A0.1 | - |
| 7 | ENBIEN0955 | EED44283.1 | - |
| 8 | VICORN1467 | ELA41393.1 | - |
| 9 | EDAEDI1466 | EJW02813.1 | - |
| 10 | VACULI1468 | ELA47012.1 | - |
| 11 | TRHOMI1469 | ELQ76742.1 | - |
| 12 | NOBOMB1471 | EOB14727.1 | - |
| 13 | NOCERA0958 | EEQ81370.1 | - |
| 14 | NESP.1463 | EHY65391.1 | - |
| 15 | NEPARI1464 | EIJ87245.1 | - |
| 16 | ENBIEN1327 | XP_002649757.1 | - |

| **AQPs** | | | |
| --- | --- | --- | --- |
| **S. NO.** | **MIPModDB ID** | **GENBANK ACCESSION** | **JGI PROTEIN ID** |
| 1 | GLINTR1048 | ACV52007.1 | - |
| 2 | GLINTR1503 | JQ412059 | - |
| 3 | MUCIRC1515 | - | 114802 |
| 4 | PUGRAM1013 | EFP76426.1 | - |
| 5 | MELARI1358 | EGG08397.1 | - |
| 6 | MELARI1359 | EGG08398.1 | - |
| 7 | MELARI1360 | EGG08399.1 | - |
| 8 | PUGRAM1012 | EFP76424.1 | - |
| 9 | MELARI1355 | EGF99782.1 | - |
| 10 | MIOSMU1499 | GAA95792 | - |
| 11 | MIOSMU1500 | GAA96083 | - |
| 12 | PHCHRY1519 | - | 136831 |
| 13 | PHCHRY1520 | - | 137867 |
| 14 | TRVERS1533 | - | 126535 |
| 15 | WOCOCO1542 | - | 120393 |
| 16 | SELACR1367 | EGN99308.1 | - |
| 17 | LABICO1505 | JQ585592 | - |
| 18 | LABICO1513 | - | 456764 |
| 19 | PIINDI1481 | CCA75077 | - |
| 20 | SPREIL1038 | CBQ67623.1 | - |
| 21 | USMAYD0878 | EAK81040.1 | - |
| 22 | MELARI1357 | EGG05742.1 | - |
| 23 | MIOSMU1501 | GAA97501 | - |
| 24 | SCSCLE0943 | EDN96810.1 | - |
| 25 | USMAYD0889 | XP_758989.1 | - |
| 26 | BLGRAM1039 | AAL77035.1 | - |
| 27 | ERGRAM0708 | - | - |
| 28 | BLGRAM0668 | - | - |
| 29 | TECLAV1502 | JF491353 | - |
| 30 | TUMELA1530 | XP_002835194 | TmeAQP1 |
| 31 | SACERE0879 | NP_015518.1 | - |
| 32 | SACERE1319 | EDN61308.1 | - |
| 33 | SACERE1342 | EGA60170.1 | - |
| 34 | SACERE1341 | EGA56339.1 | - |
| 35 | SACERE0621 | - | - |
| 36 | SACERE1343 | EGA72811.1 | - |
| 37 | SACERE1344 | EGA76526.1 | - |
| 38 | SACERE1347 | EGA84321.1 | - |
| 39 | SACERE1346 | EGA80341.1 | - |
| 40 | SACERE1326 | EEU07393.1 | - |
| 41 | SACERE1009 | ADC55259.1 | - |
| 42 | SACERE0628 | - | - |
| 43 | SACHEV0749 | - | - |
| 44 | NADAIR1396 | XP_003668934.1 | - |
| 45 | TODELB1402 | XP_003681451.1 | - |
| 46 | CAGLAB0341 | XP_444824.1 | - |
| 47 | CAGLAB0887 | XP_445420.1 | - |
| 48 | TEBLAT1428 | XP_004182287.1 | - |
| 49 | KAAFRI1420 | XP_003956520.1 | - |
| 50 | TEBLAT1427 | XP_004182011.1 | - |
| 51 | TEBLAT1424 | XP_004179361.1 | - |
| 52 | DEHANS0374 | XP_461517.1 | - |
| 53 | DEHANS1328 | XP_461517.2 | - |
| 54 | MIFARI1429 | XP_004205422.1 | - |
| 55 | ERCYMB1394 | XP_003645093.1 | - |
| 56 | ASGOSS1551 | NP_986401.2 | - |
| 57 | WIANOM1541 | - | 83661 |
| 58 | PIANGU1031 | EFW97670.1 | - |
| 59 | PIPAST0886 | XP_002492992.1 | - |
| 60 | KLLACT1330 | XP_451974.2 | - |
| 61 | SPPASS1384 | EGW34382.1 | - |
| 62 | PISTIP0883 | XP_001383665.1 | - |
| 63 | CATENU1383 | EGV65475.1 | - |
| 64 | CAALBI0882 | EAK96766.1 | - |
| 65 | CADUBL1324 | XP_002421855.1 | - |
| 66 | LOELON0951 | EDK41829.1 | - |
| 67 | CAORTH1430 | XP_003865862.1 | - |
| 68 | YALIPO0874 | XP_504854.1 | - |
| 69 | PHNODO0885 | EAT87201.1 | - |
| 70 | PYTERE1364 | XP_003303461.1 | - |
| 71 | LEMACU1024 | CBX92054.1 | - |
| 72 | COHETE1488 | - | 1086713 |
| 73 | BOFUCK0952 | EDN20800.1 | - |
| 74 | SCSCLE0954 | EDN97530.1 | - |
| 75 | TRREES1369 | EGR44423.1 | - |
| 76 | TRVIRE1538 | - | 39488 |
| 77 | TRREES1372 | EGR47949.1 | - |
| 78 | MEACRI1036 | EFY84998.1 | - |
| 79 | MEANIS1339 | EFY96089.1 | - |
| 80 | NEHAEM0949 | EEU48157.1 | - |
| 81 | GIZEAE0877 | XP_380987.1 | - |
| 82 | FUOXYS1379 | EGU79346.1 | - |
| 83 | FUOXYS1492 | - | FOXG01152P0 |
| 84 | CRNEOF0356 | XP_776423.1 | - |
| 85 | CRNEOF0357 | XP_569534.1 | - |
| 86 | CRGATT1034 | XP_003192755.1 | - |
| 87 | SPROSE1529 | - | 27302 |
| 88 | AJDERM0942 | EEQ70046.1 | - |
| 89 | GRCLAV1032 | EFX01908.1 | - |
| 90 | ASFUMI0306 | XP_746526.2 | - |
| 91 | NEFISC1316 | XP_001262474.1 | - |
| 92 | ASCLAV0933 | EAW13465.1 | - |
| 93 | ASNIDU0872 | XP_664772.1 | - |
| 94 | ASNIDU1478 | CBF78938 | - |
| 95 | PECHRY1549 | XP002558264 | - |
| 96 | ASNIGE1475 | - | 125829 |
| 97 | ASFLAV0928 | EED52312.1 | - |
| 98 | ASTERR0309 | XP_001211324.1 | - |
| 99 | ASFLAV0935 | EED52460.1 | - |
| 100 | ASORYZ1548 | XP001825978 | - |
| 101 | PABRAS0932 | EEH18381.1 | - |
| 102 | NECRAS0927 | EAA35947.2 | - |
| 103 | NETETR1368 | EGO51345.1 | - |
| 104 | SOMACR0941 | CBI58704.1 | - |
| 105 | VEALBO0937 | EEY15181.1 | - |
| 106 | VEDAHL1392 | EGY16807.1 | - |
| 107 | GLGRAM1019 | EFQ32034.1 | - |
| 108 | PEMARN0929 | EEA19904.1 | - |
| 109 | TASTIP0926 | EED14336.1 | - |
| 110 | POANSE1044 | CAP65609.1 | - |
| 111 | CHTHER1375 | EGS22578.1 | - |
| 112 | COPOSA1011 | XP_003069829.1 | - |
| 113 | MICANI0945 | EEQ29031.1 | - |
| 114 | ARGYPS1332 | XP_003174045.1 | - |
| 115 | TRTONS1349 | EGD93004.1 | - |
| 116 | TRRUBR1351 | XP_003235493.1 | - |
| 117 | GIZEAE0875 | XP_383856.1 | - |
| 118 | FUOXYS1493 | - | FOXG09680P0 |
| 119 | NEHAEM0948 | EEU37476.1 | - |
| 120 | FUOXYS1380 | EGU81510.1 | - |
| 121 | GLGRAM1016 | EFQ30366.1 | - |
| 122 | MAGRIS0880 | EDJ94979.1 | - |
| 123 | MAORYZ1405 | XP_003711197.1 | - |
| 124 | TRREES1373 | EGR48964.1 | - |
| 125 | PYTRIT0953 | EDU46834.1 | - |
| 126 | PYTERE1362 | XP_003298082.1 | - |
| 127 | CLGRAY1485 | - | 96071 |
| 128 | XAPARI1545 | - | 39605 |
| 129 | LEMACU1028 | CBY00781.1 | - |
| 130 | PYTERE1021 | EFQ86707.1 | - |
| 131 | ZYTRIT1417 | XP_003854919.1 | - |
| 132 | ZYTRIT1418 | XP_003856923.1 | - |
| 133 | BOFUCK0940 | EDN25097.1 | - |
| 134 | PHNODO0870 | EAT87291.1 | - |
| 135 | COHETE1487 | - | 106077 |
| 136 | PYTERE1023 | EFQ91930.1 | - |
| 137 | PYTRIT0947 | EDU46910.1 | - |
| 138 | PYTERE1022 | EFQ91519.1 | - |
| 139 | PYTRIT0936 | EDU49835.1 | - |
| 140 | SCSCLE0925 | EDN98188.1 | - |
| 141 | CLGRAY1484 | - | 95296 |
| 142 | ZYTRIT1410 | XP_003848252.1 | - |
| 143 | CLGRAY1483 | - | 68360 |
| 144 | COMILI1388 | EGX96316.1 | - |
| 145 | ZYTRIT1409 | XP_003847578.1 | - |
| 146 | SCSCLE0934 | EDO00223.1 | - |
| 147 | BOFUCK1552 | CCD42965.1 | - |
| 148 | COHETE1489 | - | 110751 |
| 149 | COHETE1490 | - | 1114516 |
| 150 | LEMACU1027 | CBX97751.1 | - |
| 151 | PYTRIT0931 | EDU47368.1 | - |
| 152 | PYTERE1361 | XP_003297241.1 | - |
| 153 | ZYTRIT1416 | XP_003854175.1 | - |
| 154 | BOFUCK0917 | EDN33605.1 | - |
| 155 | SCSCLE0919 | EDN95056.1 | - |
| 156 | MAGRIS0869 | EDJ99173.1 | - |
| 157 | MAORYZ1408 | XP_003719970.1 | - |
| 158 | GLGRAM1018 | EFQ31514.1 | - |
| 159 | VEDAHL1390 | EGY16427.1 | - |
| 160 | NEHAEM0920 | EEU42477.1 | - |
| 161 | GIZEAE0868 | XP_390992.1 | - |
| 162 | FUOXYS1378 | EGU77257.1 | - |
| 163 | FUOXYS1494 | - | FOXG12750P0 |

| **XIPs** | | | |
| --- | --- | --- | --- |
| **S. NO.** | **MIPModDB ID** | **GENBANK ACCESSION** | **JGI PROTEIN ID** |
| 1 | TRATRO0959 | ABDG01000060 **^#^**  [27703-27452, 27334-27112,26961-26874, 26815-26532, 26467-26289] | - |
| 2 | TRREES0961 | AAIL01000183 **^#^**  [17864-18130, 18289-18511,18709-18796, 18890-19173, 19303-19460] | - |
| 3 | MEACRI1338 | EFY92789.1 | - |
| 4 | ASTERR0962 | AAJN01000055 **^#^**  [25721-25970, 26017-26595,26663-26751] | - |
| 5 | ASNIGE1333 | XP_001390456.2 | - |
| 6 | ASFLAV0957 | EED45369.1 | - |
| 7 | ASNIGE1335 | XP_001396483.2 | - |
| 8 | PEMARN0960 | ABAR01000036 **^#^**  [47839-48091, 48166-48854] | - |
| 9 | TASTIP0965 | ABAS01000013 **^#^**  [178746-178668, 178588-178022, 177946-177870, 177773-177696] | - |
| 10 | TRVIRE0964 | ABDF01000215 **^#^**  [85889-86154, 86313-86842,86893-87062] | - |
| 11 | TRVIRE1535 | - | 42995 |
| 12 | AROLIG1385 | EGX52763.1 | - |
| 13 | COMILI1386 | EGX93694.1 | - |
| 14 | NEHAEM0956 | EEU37131.1 | - |
| 15 | GIMONI0966 | AAIM02000133 **^#^**  [47801-47522, 47424-46769] | - |
| 16 | FUOXYS0963 | AAXH01000716 **^#^**  [22960-22681, 22584-21929] | - |
| 17 | FUOXYS1377 | EGU75229.1 | - |

**^#^** Accession ID corresponds to that of whole genome shotgun sequence in NCBI. GeneMark tool was used to identify the coding regions for XIP genes. Coding regions are given in parentheses.

Gupta AB, Sankararamakrishnan R 2009. Genome-wide analysis of major intrinsic proteins in the tree plant Populus trichocarpa: characterization of XIP subfamily of aquaporins from evolutionary perspective. BMC Plant Biol 9: 134. doi: 10.1186/1471-2229-9-134
