# Supplementary material for: New subfamilies of major intrinsic proteins in fungi suggest novel transport properties in fungal channels: implications for the host-fungal interactions
Source: BMC Evol Biol. 2014 Aug 12;14:173. doi: 10.1186/s12862-014-0173-4 (PMC4236510; doi:10.1186/s12862-014-0173-4)
Supplement: Additional file 2: Table S2. — Contains inter- and intra-group average pairwise sequence identities and similarities of all fungal MIP families. [file s12862-014-0173-4-S2.doc]

**Table S2**: Average pairwise sequence identity and similarity between different fungal MIP subgroups.

|  | **α-cluster AQGPS** | **β-cluster AQGPs** | **γ1-cluster AQGPs** | **γ2-cluster AQGPs** | **δ-cluster AQGPs** | **Fps1-like AQGPs** | **Yfl054-like AQGPs**  **(FacultativeAQPs)** | **SIP-like MIPs** | **XIPs** | **AQPs** |
| --- | --- | --- | --- | --- | --- | --- | --- | --- | --- | --- |
| **α-cluster AQGPS** | 72.4  (84.5) | 48.9  (66.2) | 40.0  (59.6) | 43.8  (58.6) | 29.7  (46.8) | 44.0  (63.0) | 38.1  (55.4) | 24.3  (39.7) | 23.3  (38.5) | 27.2  (44.7) |
| **β-cluster AQGPs** |  | 59.4  (74.9) | 40.3  (60.2) | 43.2  (60.9) | 29.8  (46.0) | 41.6  (60.7) | 38.1  (57.1) | 23.3  (38.3) | 21.9  (38.4) | 26.2  (42.5) |
| **γ1-cluster AQGPs** |  |  | 61.7  (76.3) | 37.7  (59.5) | 29.3  (44.9) | 35.9  (57.1) | 36.4  (56.4) | 23.6  (39.2) | 22.4  (40.3) | 27.3  (44.2) |
| **γ2-cluster AQGPs** |  |  |  | 82.5  (92.3) | 24.2  (40.7) | 35.8  (55.9) | 31.6  (49.1) | 16.8  (32.4) | 20.0  (35.9) | 22.9  (42.2) |
| **δ-cluster AQGPs** |  |  |  |  | 50.9  (68.2) | 26.5  (44.9) | 28.4  (44.3) | 22.2  (37.6) | 22.0  (37.4) | 23.2  (40.1) |
| **Fps1-like AQGPs** |  |  |  |  |  | 63.4  (79.5) | 35.2  (54.4) | 23.3  (39.1) | 22.6  (39.8) | 23.8  (43.4) |
| **Yfl054-like AQGPs (Facultative AQPs)** |  |  |  |  |  |  | 50.9  (67.0) | 25.2  (39.0) | 22.7  (38.9) | 25.7  (43.4) |
| **SIP-like MIPs** |  |  |  |  |  |  |  | 54.4  (69.8) | 23.6  (39.9) | 27.1  (44.4) |
| **XIPs** |  |  |  |  |  |  |  |  | 50.7  (67.9) | 24.2  (40.6) |
| **AQPs** |  |  |  |  |  |  |  |  |  | 51.1  (68.7) |

Average pairwise sequence identity and similarity (values within parentheses) is given. Average pairwise sequence identity and similarity is calculated only for the transmembrane helices and regions forming half-helix Loop-B and Loop-E. Intra group average sequence identities and similarities are shown in red. Sequences were aligned using EMBOSS Needle program which forms part of EMBOSS suite of programs.Rice P, Longden I, Bleasby A 2000. EMBOSS: the European Molecular Biology Open Software Suite. Trends Genet 16: 276-277.
