# Supplementary material for: New subfamilies of major intrinsic proteins in fungi suggest novel transport properties in fungal channels: implications for the host-fungal interactions
Source: BMC Evol Biol. 2014 Aug 12;14:173. doi: 10.1186/s12862-014-0173-4 (PMC4236510; doi:10.1186/s12862-014-0173-4)
Supplement: Additional file 3: Table S3. — Shows group conservation of small and weakly polar residues at the helix-helix interface calculated for all fungal MIP subgroups. [file s12862-014-0173-4-S3.doc]

**Table S3**: Conservation of small and weakly polar residues at the helix-helix interfaces of different fungal MIP subgroups.

| **Reference**  **residues**^#^ | **α-cluster AQGPS** | **β-cluster AQGPs** | **γ1-cluster AQGPs** | **γ2-cluster AQGPs** | **δ-cluster AQGPs** | **Fps1-like AQGPs** | **Yfl054-like AQGPs(Facultative AQPs)** | **SIP-like MIPs** | **XIPs** | **AQPs** |
| --- | --- | --- | --- | --- | --- | --- | --- | --- | --- | --- |
| **T48** | 52.1 % | 41.7 % | **100 %** | **100 %** | 20.4 % | **100 %** | 66.7 % | **100 %** | **94.1 %** | **99.4 %** |
| **T55** | **100 %** | **100 %** | 40 % | **100 %** | **100 %** | **100 %** | **100 %** | 0 % | **94.1 %** | **99.4 %** |
| **A78** | **77.1 %** | 25 % | **100 %** | 60 % | **100 %** | **73.1 %** | 69.1 % | **93.8 %** | **100 %** | **99.4 %** |
| **G82** | **100 %** | **100 %** | **100 %** | **100 %** | **98.0 %** | **100 %** | **100 %** | **93.8 %** | 11.8 % | **100 %** |
| **A103** | **100 %** | **100 %** | **100 %** | **100 %** | **100 %** | **92.3 %** | **97.6 %** | **100 %** | 58.8 % | **85.3 %** |
| **G107** | **100 %** | **95.8 %** | **100 %** | **80 %** | **85.7 %** | **92.3 %** | 61.9 % | **100 %** | **100 %** | **99.4 %** |
| **G129** | **100 %** | **100 %** | **100 %** | **100 %** | **100 %** | **100 %** | **100 %** | **100 %** | **100 %** | **99.4 %** |
| **A130** | **100 %** | **100 %** | **100 %** | **100 %** | **100 %** | **100 %** | **100 %** | 12.5 % | **100 %** | **100 %** |
| **G133** | **100 %** | **100 %** | **100 %** | **100 %** | **100 %** | **100 %** | **100 %** | **100 %** | **100 %** | **100 %** |
| **T172** | **100 %** | **100 %** | **100 %** | **100 %** | 14.3 % | **100 %** | **83.3 %** | **93.8 %** | **88.2 %** | **98.1 %** |
| **S181** | **100 %** | **100 %** | **100 %** | **100 %** | **98.0 %** | **100 %** | **100 %** | 68.8 % | **100 %** | 0.61 % |
| **G203** | 0 % | 4.2 % | **100 %** | 0 % | **100 %** | 0 % | **97.6 %** | **100 %** | **100 %** | **100 %** |
| **S226** | 0 % | 0 % | 0 % | 0 % | 2.0 % | 0 % | 0 % | 50 % | **100 %** | **99.4 %** |
| **G248** | **95.8 %** | **100 %** | **100 %** | **100 %** | 55.1 % | **80.8 %** | **100 %** | **100 %** | **100 %** | **100 %** |
| **G252** | **100 %** | **100 %** | **100 %** | **100 %** | **100 %** | **100 %** | **100 %** | **100 %** | **94.1 %** | **99.4 %** |
| **A253** | **100 %** | **95.8 %** | **100 %** | **100 %** | **89.8 %** | **96.2 %** | **100 %** | **100 %** | **100 %** | **99.4 %** |
| **A256** | **100 %** | **100 %** | **100 %** | **100 %** | **100 %** | **100 %** | **100 %** | **100 %** | **0 %** | **100 %** |

^#^ Reference residues correspond to that of plant aquaporin SoPIP2;1 (PDB ID: 1Z98).

Group conservation of small and weakly polar residues (Gly, Ala, Ser, Thr and Cys) is given. Positions having conservation greater than 70% are shown in bold. . Positions having conservation less than 70% are given in red color.
