# Supplementary material for: New subfamilies of major intrinsic proteins in fungi suggest novel transport properties in fungal channels: implications for the host-fungal interactions
Source: BMC Evol Biol. 2014 Aug 12;14:173. doi: 10.1186/s12862-014-0173-4 (PMC4236510; doi:10.1186/s12862-014-0173-4)
Supplement: Additional file 6: Table S4. — Contains list of pathogenic fungi that have at least one SIP-like MIP channel. [file s12862-014-0173-4-S6.doc]

**Table S4**: List of pathogenic fungi belonging SIP-like cluster:

| **Organism** | **Clinical manifestations** |
| --- | --- |
| *Encephalitozooncuniculi* | An opportunistic human pathogen. Causes several gastrointestinal diseases such as enteritis, diarrhea in immunocompromised patients. |
| *Encephalitozoonintestinalis* | An opportunistic human pathogen. Causes several gastrointestinal diseases such as enteritis, diarrhea in immunocompromised patients. |
| *Encephalitozoonromaleae* | Entomopathogenic fungi, infects eastern lubber grasshopper (*Romaleamicroptera*). |
| *Encephalitozoonhellem* | Human pathogen, Cause Of Ocular, Respiratory, Urogenital, And Systemic Infections. |
| *Nosemaceranae* | Entomopathogenic fungi, Honey bee (*Apismellifera*) pathogen. |
| *Nosemabombycis* | Entomopathogenic fungi, causes pébrine disease in silkworms. |
| *Nematocidaparisii* | Entomopathogenic fungi, infects C. Elegans. |
| *Edhazardiaaedis* | Entomopathogenic fungi, infects yellow fever parasite *Aedesaegypti*. *E. aedis* has been intensively studied as a biological control agent for *A. aegypti*. |
| *Vavraiaculicisfloridensis* | Entomopathogenic fungi, infects malaria parasite *Anopheles gambiae*. |
| *Trachipleistophorahominis* | An opportunistic human pathogen. Cause myositis in immunocompromised patients. |
| *Enterocytozoonbieneusi* | An opportunistic human pathogen. Associated with gastrointestinal disease in humans. Most commonly cause chronic diarrhea. |
| *Vittaformacorneae* | Human pathogen. Causes keratitis (corneal infections) in both immunocompromised and healthy persons. |

**References:**

Andreadis TG 1994. Host range tests with Edhazardia aedis (Microsporida: Culicosporidae) against northern Nearctic mosquitoes. J Invertebr Pathol 64: 46-51. doi: 10.1006/jipa.1994.1067

Bargielowski I, Koella JC 2009. A possible mechanism for the suppression of Plasmodium berghei development in the mosquito Anopheles gambiae by the microsporidian Vavraia culicis. PLoS One 4: e4676. doi: 10.1371/journal.pone.0004676

Cuomo CA, Desjardins CA, Bakowski MA, Goldberg J, Ma AT, Becnel JJ, Didier ES, Fan L, Heiman DI, Levin JZ, Young S, Zeng Q, Troemel ER 2012. Microsporidian genome analysis reveals evolutionary strategies for obligate intracellular growth. Genome Res 22: 2478-2488. doi: 10.1101/gr.142802.112

Field AS, Marriott DJ, Milliken ST, Brew BJ, Canning EU, Kench JG, Darveniza P, Harkness JL 1996. Myositis associated with a newly described microsporidian, Trachipleistophora hominis, in a patient with AIDS. J Clin Microbiol 34: 2803-2811.

Franzen C, Muller A 2001. Microsporidiosis: human diseases and diagnosis. Microbes Infect 3: 389-400.

Higes M, Meana A, Bartolome C, Botias C, Martin-Hernandez R 2013. Nosema ceranae (Microsporidia), a controversial 21st century honey bee pathogen. Environ Microbiol Rep 5: 17-29. doi: 10.1111/1758-2229.12024

Johny S, Larson TM, Solter LF, Edwards KA, Whitman DW 2009. Phylogenetic characterization of Encephalitozoon romaleae (Microsporidia) from a grasshopper host: relationship to Encephalitozoon spp. infecting humans. Infect Genet Evol 9: 189-195. doi: 10.1016/j.meegid.2008.10.010

Notermans DW, Peek R, de Jong MD, Wentink-Bonnema EM, Boom R, van Gool T 2005. Detection and identification of Enterocytozoon bieneusi and Encephalitozoon species in stool and urine specimens by PCR and differential hybridization. J Clin Microbiol 43: 610-614. doi: 10.1128/JCM.43.2.610-614.2005

Pan G, Xu J, Li T, Xia Q, Liu SL, Zhang G, Li S, Li C, Liu H, Yang L, Liu T, Zhang X, Wu Z, Fan W, Dang X, Xiang H, Tao M, Li Y, Hu J, Li Z, Lin L, Luo J, Geng L, Wang L, Long M, Wan Y, He N, Zhang Z, Lu C, Keeling PJ, Wang J, Xiang Z, Zhou Z 2013. Comparative genomics of parasitic silkworm microsporidia reveal an association between genome expansion and host adaptation. BMC Genomics 14: 186. doi: 10.1186/1471-2164-14-186
