# Supplementary material for: New subfamilies of major intrinsic proteins in fungi suggest novel transport properties in fungal channels: implications for the host-fungal interactions
Source: BMC Evol Biol. 2014 Aug 12;14:173. doi: 10.1186/s12862-014-0173-4 (PMC4236510; doi:10.1186/s12862-014-0173-4)
Supplement: Additional file 7: Table S5. — Contains list of plant pathogenic fungi that have at least one member from the δ subgroup of AQGPs. [file s12862-014-0173-4-S7.doc]

**Table S5**: List of pathogenic fungi belonging to -cluster AQGPs:

| **Organism** | **Clinical manifestations** |
| --- | --- |
| Botryotiniafuckeliana | Causal agent of the grey mold disease. |
| Dothistromaseptosporum | Causes needle blight in pine trees. |
| Fomitiporiamediterranea | Causes Esca disease in *VitisVinifera* (wine grape). |
| Gaeumannomycesgraminis | Causes Crown and sheath rot disease in rice . |
| Magnaportheoryzae | Causes blast of rice disease in rice and blast of wheat disease in wheat . |
| Marssoninabrunnea | Causes Marssonina leaf spot, one of the most devastating diseases, in Populus. |
| Melampsoralarici-populina | Causes rust, one of the most devastating diseases, in Populus |
| Mixiaosmundae | Infect ferns . |
| Mycosphaerellapopulorum | Causes leaf spots and cankers on poplars . |
| Pseudocercosporafijiensis | Causes Black Sigatoka (black leaf streak) disease of banana . |
| Sclerotiniasclerotiorum | Sclerotinia disease, Sclerotinia wilt, Sclerotinia rot, stem blight, head rot . |
| Sporisoriumreilianum | Causes Head smut in Poaceae family of plants . |
| Taphrinadeformans | Causes peach leaf curl disease in plants . |
| Ustilagomaydis | Causes corn smut disease in *Zea Mays*. |
| Ustilagohordei | Causes smut disease in Poaceae family of plants . |
| Verticilliumalbo-atrum | Causes verticillium wilt in economically important plants such as olive, maple, potatoes, cotton etc. |
| Verticilliumdahliae | Causes verticillium wilt in economically important plants such as olive, maple, potatoes, cotton etc. |
| Wolfiporiacocos | Infectsconifer and hardwood plants. |
| Zymoseptoriatritici | Causes leaf spot or speckled leaf blotch of wheat. |

**References:**
