# Supplementary figures and images for: New subfamilies of major intrinsic proteins in fungi suggest novel transport properties in fungal channels: implications for the host-fungal interactions
Source: BMC Evol Biol. 2014 Aug 12;14:173. doi: 10.1186/s12862-014-0173-4 (PMC4236510; doi:10.1186/s12862-014-0173-4)

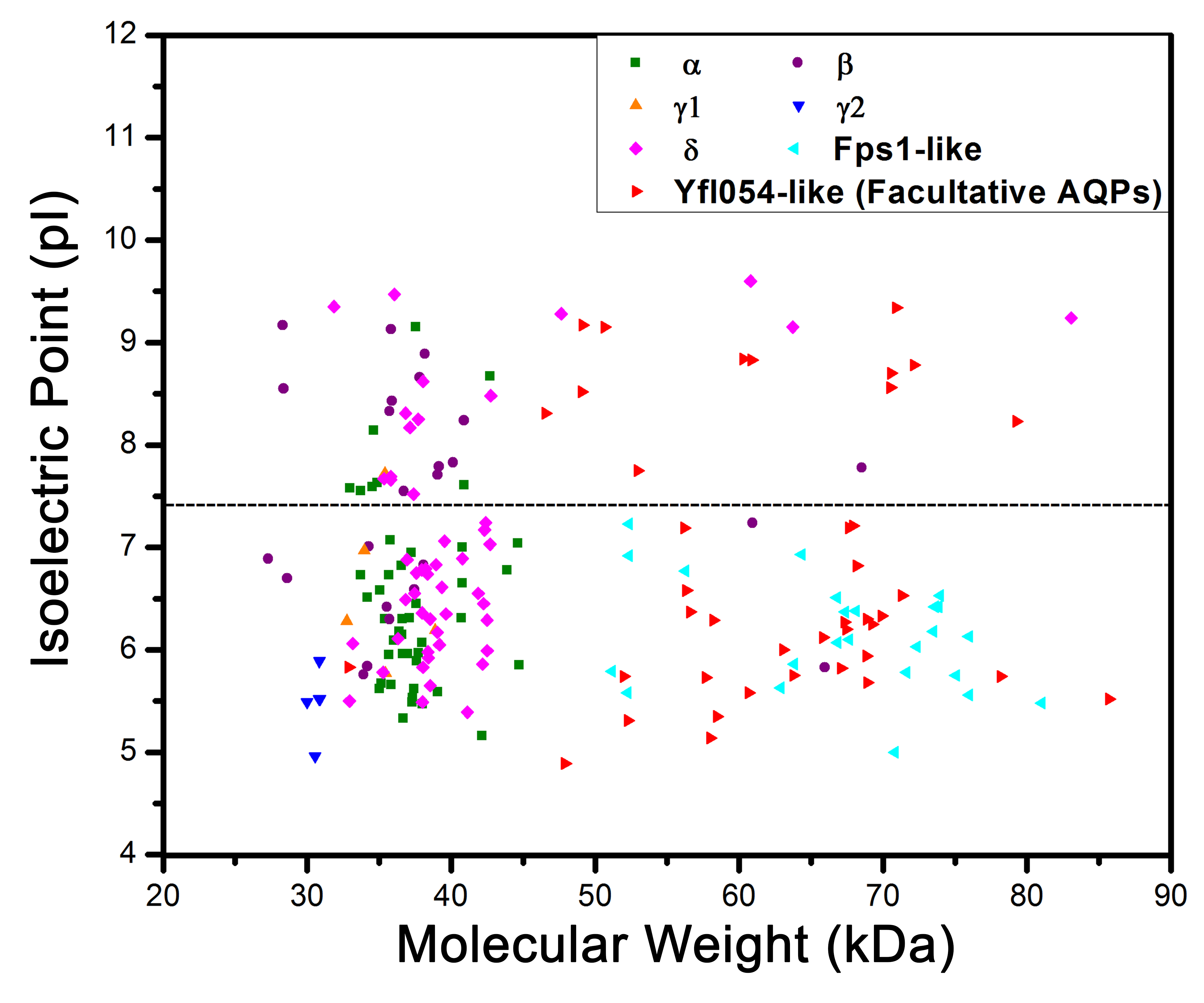

Supplement: Additional file 4: Figure S1. — Relationship between isoelectric point and molecular weight for all the fungal AQGP groups. [file s12862-014-0173-4-S4.tiff]

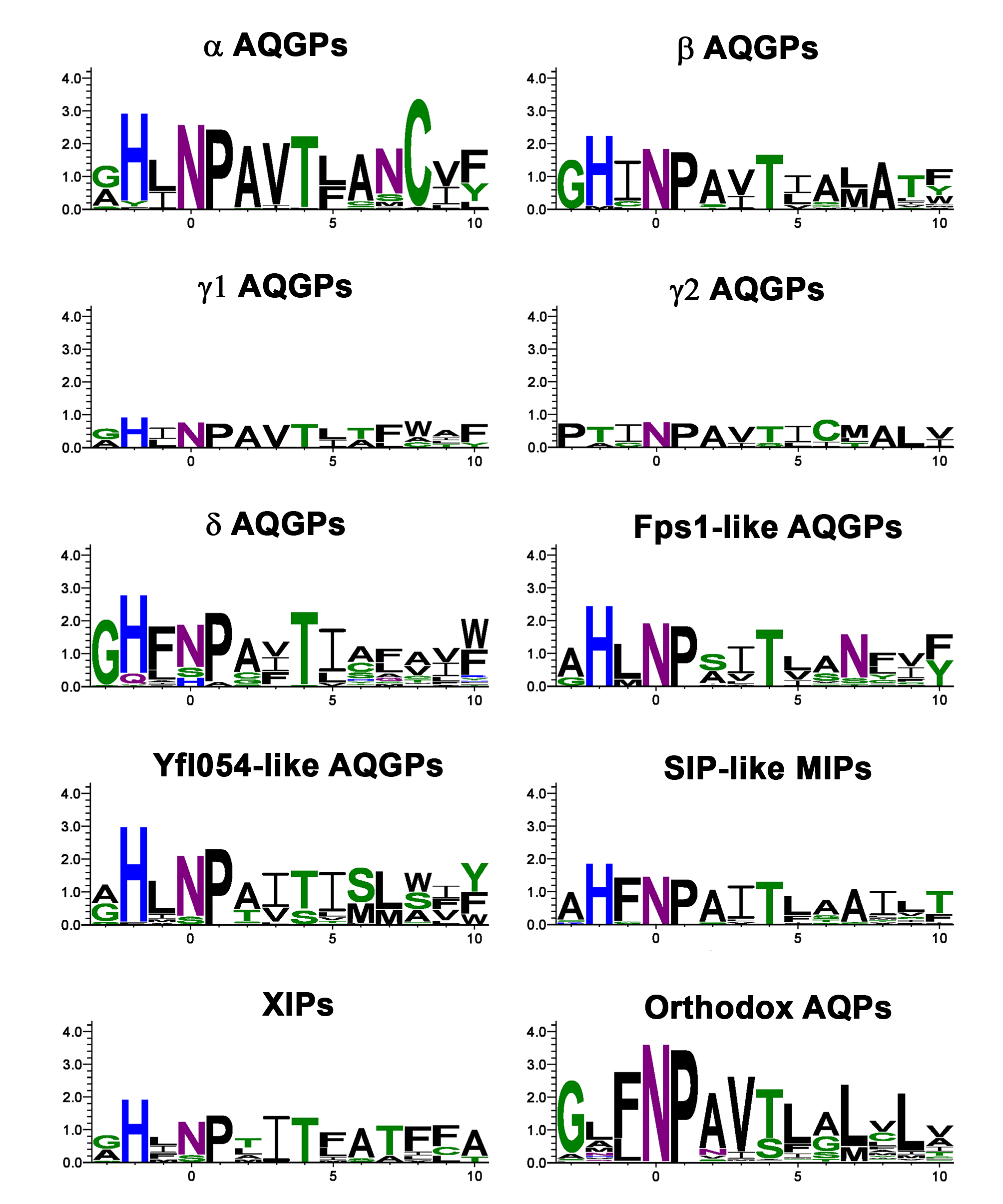

Supplement: Additional file 5: Figure S2. — Contains sequence logos produced for Loop B region for different fungal MIP groups. [file s12862-014-0173-4-S5.tiff]
